# Accuracy of Fitbit Devices: Systematic Review and Narrative Syntheses of Quantitative Data

**DOI:** 10.2196/10527

**Published:** 2018-08-09

**Authors:** Lynne M Feehan, Jasmina Geldman, Eric C Sayre, Chance Park, Allison M Ezzat, Ju Young Yoo, Clayon B Hamilton, Linda C Li

**Affiliations:** ^1^ Department of Physical Therapy University of British Columbia Vancouver, BC Canada; ^2^ Arthritis Research Canada Richmond, BC Canada; ^3^ School of Population and Public Health University of British Columbia Vancouver, BC Canada; ^4^ BC Children’s Hospital Research Institute Vancouver, BC Canada

**Keywords:** wearable activity tracker, accuracy, Fitbit, steps, sleep, energy expenditure, distance, time in activity, systematic review, fitness trackers, data accuracy, energy metabolism, review

## Abstract

**Background:**

Although designed as a consumer product to help motivate individuals to be physically active, Fitbit activity trackers are becoming increasingly popular as measurement tools in physical activity and health promotion research and are also commonly used to inform health care decisions.

**Objective:**

The objective of this review was to systematically evaluate and report measurement accuracy for Fitbit activity trackers in controlled and free-living settings.

**Methods:**

We conducted electronic searches using PubMed, EMBASE, CINAHL, and SPORTDiscus databases with a supplementary Google Scholar search. We considered original research published in English comparing Fitbit versus a reference- or research-standard criterion in healthy adults and those living with any health condition or disability. We assessed risk of bias using a modification of the Consensus-Based Standards for the Selection of Health Status Measurement Instruments. We explored measurement accuracy for steps, energy expenditure, sleep, time in activity, and distance using group percentage differences as the common rubric for error comparisons. We conducted descriptive analyses for frequency of accuracy comparisons within a ±3% error in controlled and ±10% error in free-living settings and assessed for potential bias of over- or underestimation. We secondarily explored how variations in body placement, ambulation speed, or type of activity influenced accuracy.

**Results:**

We included 67 studies. Consistent evidence indicated that Fitbit devices were likely to meet acceptable accuracy for step count approximately half the time, with a tendency to underestimate steps in controlled testing and overestimate steps in free-living settings. Findings also suggested a greater tendency to provide accurate measures for steps during normal or self-paced walking with torso placement, during jogging with wrist placement, and during slow or very slow walking with ankle placement in adults with no mobility limitations. Consistent evidence indicated that Fitbit devices were unlikely to provide accurate measures for energy expenditure in any testing condition. Evidence from a few studies also suggested that, compared with research-grade accelerometers, Fitbit devices may provide similar measures for time in bed and time sleeping, while likely markedly overestimating time spent in higher-intensity activities and underestimating distance during faster-paced ambulation. However, further accuracy studies are warranted. Our point estimations for mean or median percentage error gave equal weighting to all accuracy comparisons, possibly misrepresenting the true point estimate for measurement bias for some of the testing conditions we examined.

**Conclusions:**

Other than for measures of steps in adults with no limitations in mobility, discretion should be used when considering the use of Fitbit devices as an outcome measurement tool in research or to inform health care decisions, as there are seemingly a limited number of situations where the device is likely to provide accurate measurement.

## Introduction

Commercially available wearable activity trackers have grown rapidly in popularity since their introduction just over a decade ago [1]. While the technologies behind them are quickly and continuously changing, in general they are small devices that are commonly worn on the wrist or attached to clothing. They aim to provide the user with real-time feedback on various aspects of daily activities, such as number of steps taken, energy expenditure, time spent asleep, and time spent in different levels of activity. They also typically provide personal goal-setting options, summary data, and visualizations through synchronization with interactive mobile- and computer-based apps, as well as opportunities to connect to social media and other health and fitness apps. These devices are aimed primarily at health- and fitness-conscious consumers and are designed to motivate and offer support to individuals to self-monitor and increase their daily physical activity.

Fitbit (Fitbit Inc, San Francisco, CA, USA), one of the most popular commercial wearable activity trackers, holds approximately 20% of the market share for wearable tracking devices, with more than 63 million devices sold worldwide in the last 10 years [2]. In 2017, the company sold 15 million devices and had 25 million active users [2]. The Classic model was introduced in 2009 as a clip-on device to be worn on the torso; new models of clip-on devices became commercially available in 2011 with the introduction of the Ultra, Zip, and One models. In 2013, Fitbit introduced a line of wristband activity trackers: Force, Flex (2), Charge (2, HR), and Alta (HR).

Fitbit devices use a microelectronic triaxial accelerometer to capture body motion in 3-dimensional space, with these motion data analyzed using proprietary algorithms to identify patterns of motion to identify daily steps taken, energy expenditure, sleep, distance covered, and time spent in different intensity of activities. Although designed as a consumer product to help motivate individuals to be physically active, Fitbit devices are becoming increasingly popular as measurement tools in physical activity and health promotion research and are also commonly used to inform patient–health professional interactions [3-7]. Between 2011 and 2017, a total of 171 clinical trials registered at ClinicalTrials.gov used Fitbit as an outcome measurement tool; 97 of those were registered in the last 3 years [8]. Most of the registered trials identified number of steps taken as the outcome of interest, followed, in order, by time in activity, sleep, energy expenditure, and distance covered.

Fitbit devices, and particularly the wrist-worn devices, have demonstrated dependability, durability, and acceptability [9,10]. A 2015 systematic review by Evenson et al examined the “validity and reliability of...[Fitbit devices] and their ability to estimate steps, distance, physical activity, energy expenditure, and sleep” [11]. They concluded that Fitbit devices were moderately associated with criterion reference devices for measures of steps, sleep, and distance, with associations varying from poor to moderate with criterion reference devices for measures of energy expenditure and time in activity [11]. They also found that Fitbit had a high interdevice reliability for all outcome measures. In addition, the review provided some data for measurement accuracy; however, it did not comprehensively examine device measurement accuracy or study quality. Measurement accuracy, or how close to “true” the measured value is, is an important consideration, as Fitbit devices are being used as an outcome measurement tool in research and to inform health care decisions [12,13]. Therefore, the purpose of this review was to systematically examine and report the accuracy of measures derived from the triaxial accelerometry data in Fitbit devices—that is, measures of steps, energy expenditure, sleep, distance, and time in activity—when used by adults in controlled and free-living settings.

## Methods

### Search Strategy

We conducted an electronic literature search of the PubMed, EMBASE, CINAHL, and SPORTDiscus databases, with an additional supplementary search conducted via Google Scholar. Keywords within each database search included variations on the terms Fitbit AND Accuracy (accura*) OR Validity / Validation (valid*) OR Comparison / Comparative (compar*) OR Relationship (relation*) OR Association (associa*) Or Equivalence (equival*) OR Agreement (Multimedia Appendix 1). We applied a language filter to limit results to English and a date limitation from January 1, 2011 (Fitbit devices were not commercially available prior to this date) to October 31, 2017 (the end date for our search). We applied no further search limits or filters. We also hand searched reference lists of the included studies for potentially eligible studies.

### Study Selection and Eligibility Criteria

We screened all citations, removed duplicates, and assessed the remaining titles and abstracts for potential eligibility. All potentially eligible citations were retrieved for full-text review by 2 independent reviewers (JYY, JG) with disagreements resolved through consensus. Initial inclusion criteria were original research studies, published in a full, short, or letter format in English in peer-reviewed journals. We verified journal peer-review status using a Web-based serial directory database search (ULRICHSWEB, ProQuest LLC, Ann Arbor, MI, USA). The studies also had to include or separately report data for adults (≥18 years old) and examine measurement accuracy for one or more of the following outcome domains: steps, energy expenditure, time in activity, distance, or sleep. Studies could be conducted in any controlled-testing (ie, using a standardized testing protocol) or free-living (ie, during usual daily activity) setting and could include individuals living with any health, disease, or mobility or functional status. Studies examining accuracy in controlled settings had to compare a Fitbit measure against a predefined reference-standard criterion measure, whereas studies conducted in free-living settings had to compare the Fitbit measure against a predefined research-standard criterion measure (Multimedia Appendix 2). To be included in the final review, studies had to have extractable data for one or more of the following accuracy analyses: group mean or percentage differences, mean or median absolute percentage error (MAPE), or level-of-agreement analyses [14]. We did not contact authors if these data were not reported in the publication. We excluded studies (or comparisons) if the accuracy evaluations were conducted on 10 or fewer participants. We also excluded studies (or comparisons) if they examined heart rate accuracy, as heart rate measurement is not derived from accelerometry data.

### Data Extraction

Data were extracted and checked for accuracy by a second independent reviewer (JYY, JG, AME, LMF) with discrepancies resolved through discussion and consensus. Data extracted included study, participant, and Fitbit device characteristics, as well as details about the study setting, outcomes examined, and reference criterion used (Multimedia Appendix 2). Group mean or percentage difference values for the Fitbit device and criterion groups were extracted for all accuracy comparisons reported in each study. If group percentage difference was not reported, we calculated group percentage error ([Fitbit_mean_–Criterion_mean_] / Criterion_mean_×100) to allow for a common unit of measure (rubric) for comparison of accuracy measures within and across outcome domains (Multimedia Appendix 3). We also extracted reported MAPE or level-of-agreement accuracy data when available.

### Risk-of-Bias Assessment

All articles were independently assessed for risk of bias by 2 independent reviewers (JG, CP) using a modification of the validation subscale from the checklist for assessing the methodological quality of studies on measurement properties of health status measurement instruments (Consensus-Based Standards for the Selection of Health Status Measurement Instruments [COSMIN]) [15]. All discrepancies were resolved by discussion and consensus, or by a third independent reviewer (LMF). Quality evaluation included 5 design or methodology components (percentage missing data, missing data management, adequate sample size, acceptable criterion comparison, design or methodological flaws) and one analysis component (acceptable accuracy analyses). We rated each dimension as excellent, good, fair, or poor quality based on a priori modifications to the COSMIN validation subscale scoring criteria appropriate for accuracy studies (Multimedia Appendix 4) [16].

### Data Handling

We sorted each accuracy comparison into one of the following outcome domains: (1) steps, (2) energy expenditure, (3) sleep, (4) time in activity, or (5) distance. Within each domain, we coded individual accuracy comparisons to identify testing parameters that may influence measurement accuracy, such as variations in the testing environment(s), placement of device(s), or variations in the type of ambulation or activity or task examined (Multimedia Appendix 5). All coding was independently reviewed by a second reviewer with discrepancies resolved through discussion and consensus (LMF, JG).

### Syntheses

Given the diversity of outcomes reported and the variety of testing conditions under which accuracy measures were examined and reported across and within different studies, we were unable to conduct meta-analyses. As an alternative, and as recommended by the UK Economic and Social Research Council guidelines for conducting and reporting narrative syntheses, we conducted a narrative synthesis of quantitative data, where we explored measurement accuracy within each outcome domain (ie, steps, energy expenditure, sleep, time in activity, and distance) using group percentage difference as the common rubric for measurement error comparisons [17,18]. We performed descriptive analyses for frequency (number and percentage) of percentage error comparisons that were within and outside predefined cutoff points for measurement accuracy in controlled or free-living settings. We also explored potential trends for direction of measurement error (ie, potential measurement bias) by defining a point estimation for both mean and median percentage error, with negative values indicating a trend for Fitbit device underestimation compared with the criterion device. In addition, we explored measurement error dispersion by defining the range (maximum–minimum) for percentage error measures. Given the diversity of testing conditions, we conducted further secondary exploratory analyses for comparisons of steps and energy expenditure accuracy in controlled settings to examine the potential influence of different testing parameters such as variations in body placement, ambulation speed, or variations in the type of activity on measurement accuracy. We completed these secondary exploratory analyses only when there were 10 or more accuracy comparisons within each subgroup.

We provide summaries for all descriptive analyses in tabular formats. For selected secondary subanalyses, we also provide modified scatter plots depicting the distribution of accuracy comparisons for group percentage error, color coded by variations in testing parameters, to allow for visual interpretation of how measurement error may be influenced by variations in testing parameters.

We focused our interpretation of measurement accuracy based on predefined acceptable limits for measurement accuracy in controlled settings as a percentage difference of ±3% and acceptable limits for relative accuracy in free-living settings as a percentage difference of ±10% [19-22]. We completed all descriptive analyses and plots using SAS version 9.4 software (SAS Institute Inc).

In the review, we included accuracy studies not reporting data to allow for the examination of group percentage measurement error if they reported MAPE or level-of-agreement data. These studies were included in the syntheses of study characteristics and the risk-of-bias assessment. As well, we provide narrative summaries for how the reported accuracy from these studies may or may not be consistent with our evaluation of percentage measurement error.

## Results

### Study Selection

**Figure 1:**
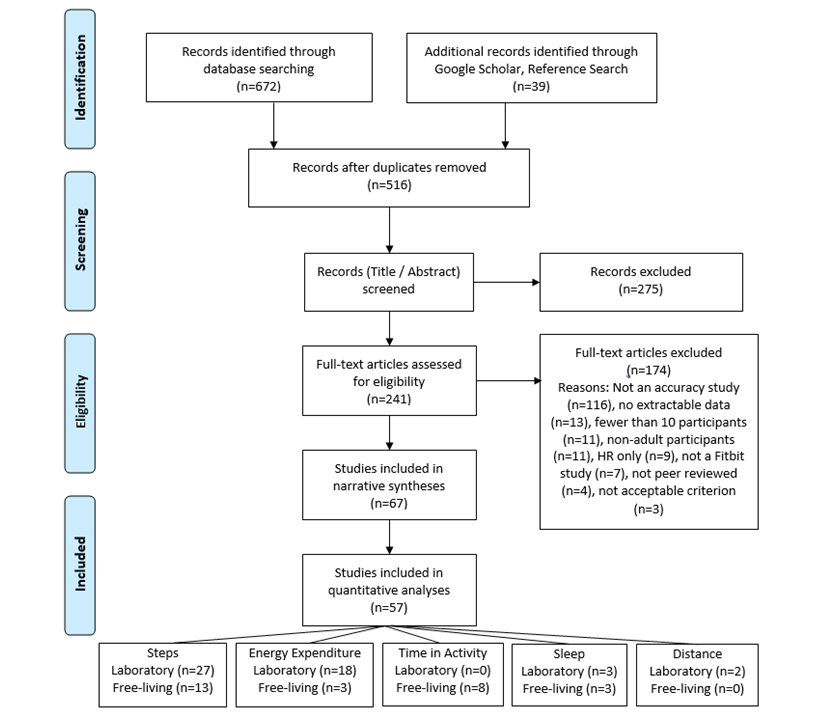
Preferred Reporting Items for Systematic Reviews and Meta-Analyses (PRISMA) flow chart.

We identified 711 citations, of which we screened 516 titles and abstracts for potential eligibility after removing duplicates. Following screening, we excluded 275 titles, with the remaining 241 full-text reviewed. After full-text review, we subsequently excluded 174 articles. A total of 67 studies met the final inclusion criteria, with 57 providing adequate data for inclusion in the quantitative analyses. Of these, 40 studies investigated step count (laboratory: n=27; free-living settings: n=13), 21 addressed energy expenditure (laboratory: n=18; free-living settings: n=3), 8 examined time spent in different intensities of activity in free-living settings, 6 examined sleep measurements (laboratory: n=3; free-living settings: n=3), and 2 examined distance walked in a controlled testing environment (Figure 1) [23].

### Study and Participant Characteristics

Publication dates varied from 1 publication in each of 2012 and in 2013, to 8 publications in each of 2014 and in 2015, with 49 studies published in or after 2016. Publications were from 11 countries across North America, Western Europe, South Asia, and Australia. The largest number of publications were from the United States (n=39), followed by Australia (n=9) and Canada (n=5). Of the 67 publications, 61 were full research articles, 5 were short reports, and 1 was a letter to the editor (Multimedia Appendix 6).

The 67 studies comprised a total of 2441 participants, with the mean number being 36 (SD 25), varying from 12 to 166. Of the 61 studies reporting age, the mean age of participants was 37 (SD 18) years, varying from 21 to 84 years. Of the 65 studies reporting sex, 53.95% (1251/2319) of the participants were female. A total of 55 studies included only healthy participants, with the remaining 12 including participants living with a variety of chronic diseases or mobility limitations, or both (Multimedia Appendix 6). Studies used several models of Fitbit devices, including the Ultra, Classic, Zip, or One worn on the torso (waist, hip, or chest), the Flex, Charge HR, Force, or Surge worn on the wrist, and the One worn on the ankle.

### Risk of Bias

We rated the vast majority of the 67 studies as excellent or good for study design, reporting of missing data, and use of an acceptable reference criterion measure (Multimedia Appendix 7). For 34 studies (43 accuracy comparisons), it was unclear how missing data were handled in the analyses (Multimedia Appendix 7). We did not exclude these accuracy comparisons from the descriptive analyses of percentage measurement error based on this criterion. However, we did exclude 21 accuracy comparisons in the descriptive analyses, as the Fitbit versus criterion group mean or percentage differences were not reported (Multimedia Appendix 7). Rather than excluding these accuracy comparisons (or studies) completely from the review, we provide a narrative summary for how the reported MAPE or level-of-agreement accuracy data may or may not be consistent with our exploration of percentage measurement error.

**Figure 2:**
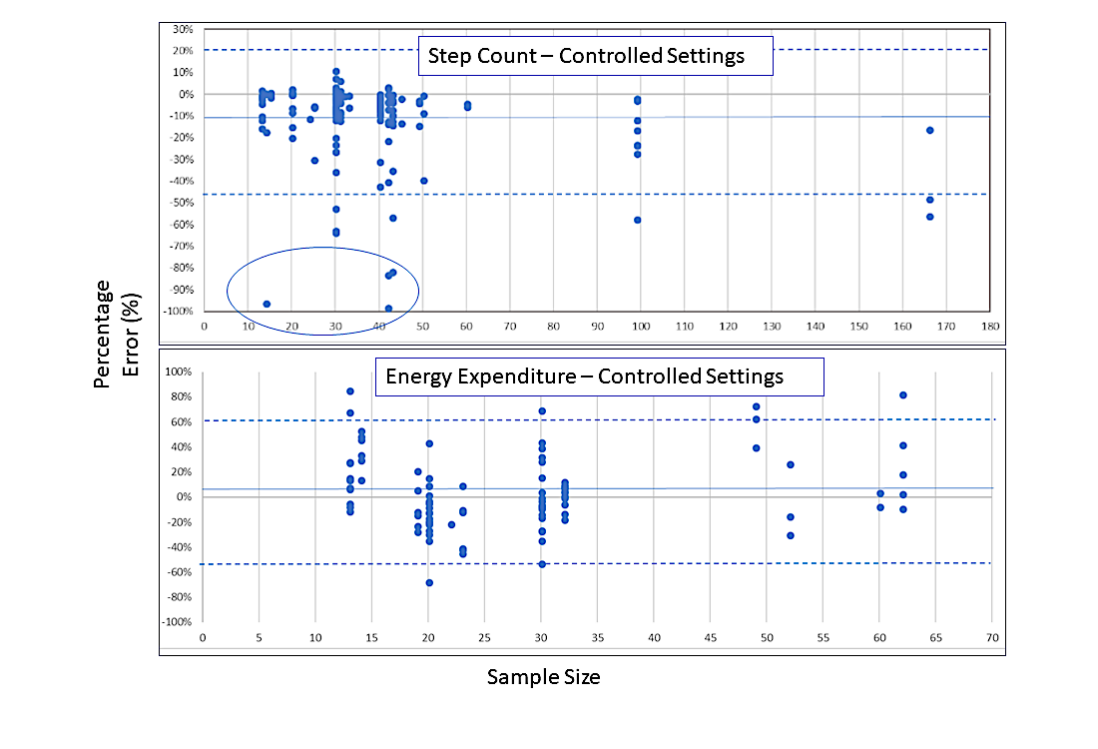
Percentage error distribution by sample size. Top: Step count in controlled settings. The blue oval indicates extreme outliers (n=4 comparisons). Bottom: Energy expenditure in controlled settings. Solid blue lines indicate mean error estimation. Dotted blue lines indicate 95% CI.

We also did not exclude 55 studies (85 accuracy comparisons) rated fair or poor for sample size (<50 participants), as there were only 2 studies with 100 or more participants (excellent rating) and 10 with 50 to 99 participants (good rating) (Multimedia Appendix 7). Rather, we excluded studies (or accuracy comparisons) with 10 or fewer participants. As well, for step count and energy expenditure in controlled settings, we explored the potential for bias based on sample size by exploring the dispersion of group percentage error across different sample sizes using modified scatter plots (Figure 2). In these exploratory analyses, we saw no apparent systematic bias for measurement error, other than a slight tendency for extreme underestimation of steps in 4 comparisons from 2 studies with fewer than 50 participants. However, when we explored these extreme outliers further, we determined that they were likely true reflections of a greater tendency for underestimation of step count during very slow walking activities when the device was worn on the torso, rather than due to small sample size. Therefore, we included all percentage error accuracy comparisons, independent of sample size, in our descriptive analyses.

### Step Count

A total of 27 studies (191 accuracy comparisons) examined Fitbit device step measurements compared with a reference-standard criterion of direct observation and counting of steps in a controlled setting (Multimedia Appendix 3) [12,24-49]. Of these, 21 studies recruited healthy adults with a mean age of 37.2 (SD 18.3) years; the remaining 6 recruited adults living with limited mobility or chronic disease with a mean age of 64.8 (SD 14.8) years. Fitbit devices were worn on the torso, wrist, or ankle. Across the 191 accuracy comparisons examining step count in controlled settings, 46% (n=88) were within a ±3% measurement error, 51% (n=97) were below a –3% measurement error, and 3% (n=6) were above a 3% measurement error, with an overall tendency for Fitbit devices to underestimate steps (estimated mean [median] difference of –9% [–3%]) (Multimedia Appendix 8 and Figure 2).

When we further explored factors potentially influencing step count accuracy, we observed that accuracy of step count in controlled settings seemed to vary with speed of ambulation (jog, normal, self-paced, slow, or very slow) [50,51], with body placement (torso, wrist, or ankle), and with variations in how the body moved during the activity (normal, constrained, variable, or exaggerated) (Multimedia Appendix 8). Constrained body motion throughout the task or activity could be due to, for example, a disease-related mobility limitation, walking with a walking aide, or pushing a stroller while walking. Levels of body motion could have varied while performing a series of different simulated household tasks or doing simulated agility-dependent sporting activities. Exaggerated motions could have occurred when the device was worn on the wrist during simulated household or sporting activities that involved exaggerated arm motions.

**Figure 3:**
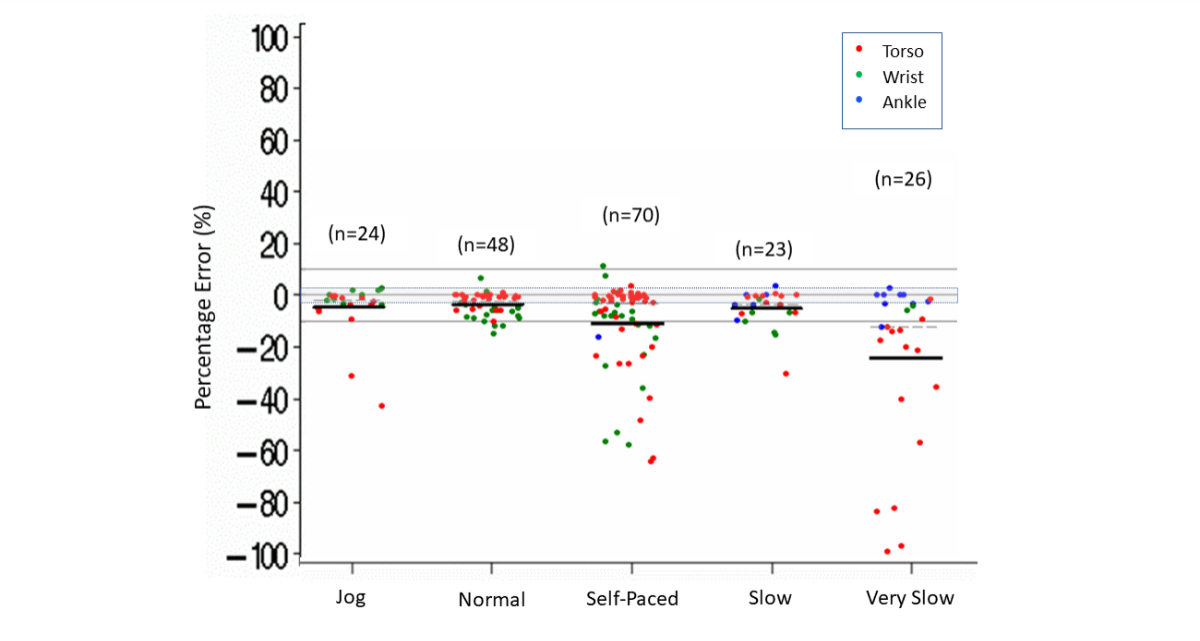
Step count percentage error in controlled settings. Speed (jog, normal, self-paced, slow, very slow) by body placement (torso, wrist, ankle) of the Fitbit device. Dark lines indicate mean (horizontal). Dashed lines indicate median (horizontal). Gray shading indicates ±3% measurement error.

Within the different speeds of ambulation, measurement error was within ±3% more than 50% of the time for jogging (14/24) or normal (25/48) ambulation speeds. More than 50% of the time, measurement error was below –3% for self-paced (35/70), slow (12/23), and very slow (19/26) ambulation speeds. Within each ambulation speed, Fitbit tended to underestimate step counts (mean [median] error estimations varying from –24% [–12%] to –4% [–2%]) (Multimedia Appendix 8 and Figure 3).

**Figure 4:**
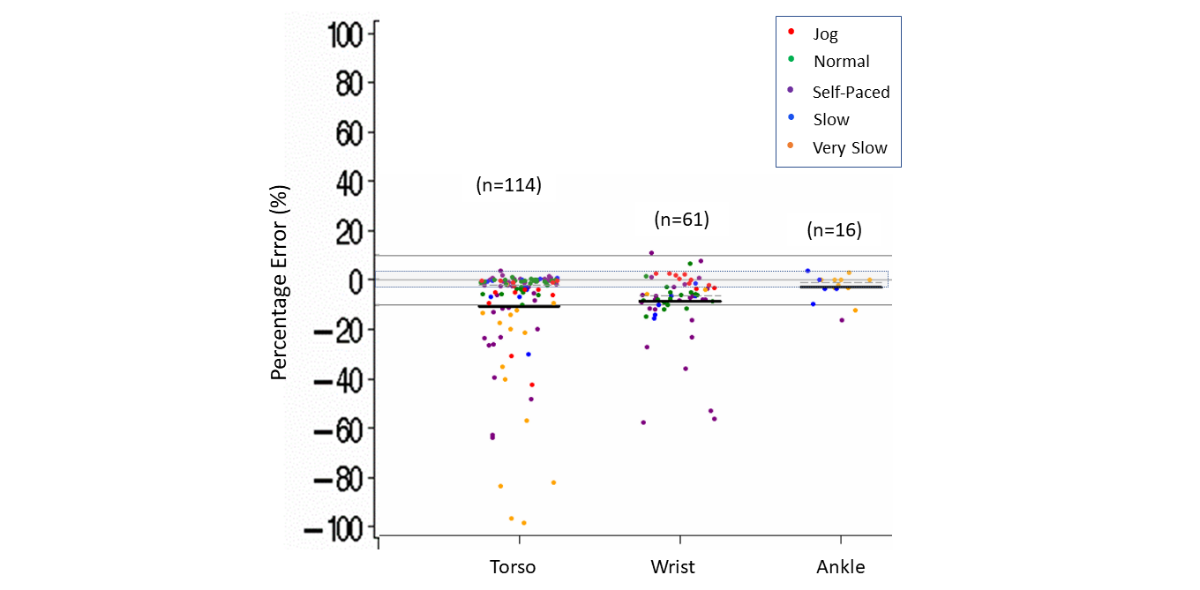
Step count percentage error in controlled settings. Body placement (torso, wrist, ankle) of the Fitbit device by speed (jog, normal, self-paced, slow, very slow). Dark lines indicate mean (horizontal). Dashed lines indicate median (horizontal). Gray shading indicates ±3% measurement error.

Within the different body placements for the device, measurement error was within ±3% more than 50% of the time for comparisons with torso (65/114) or ankle (8/16) placement, whereas 70% (43/61) of the time measurement error was below –3% for wrist placement. Within each body placement, Fitbit tended to underestimate steps (estimated mean [median] errors varying from a –11% [–2%] to –3% [–1%]) (Multimedia Appendix 8 and Figure 4).

**Figure 5:**
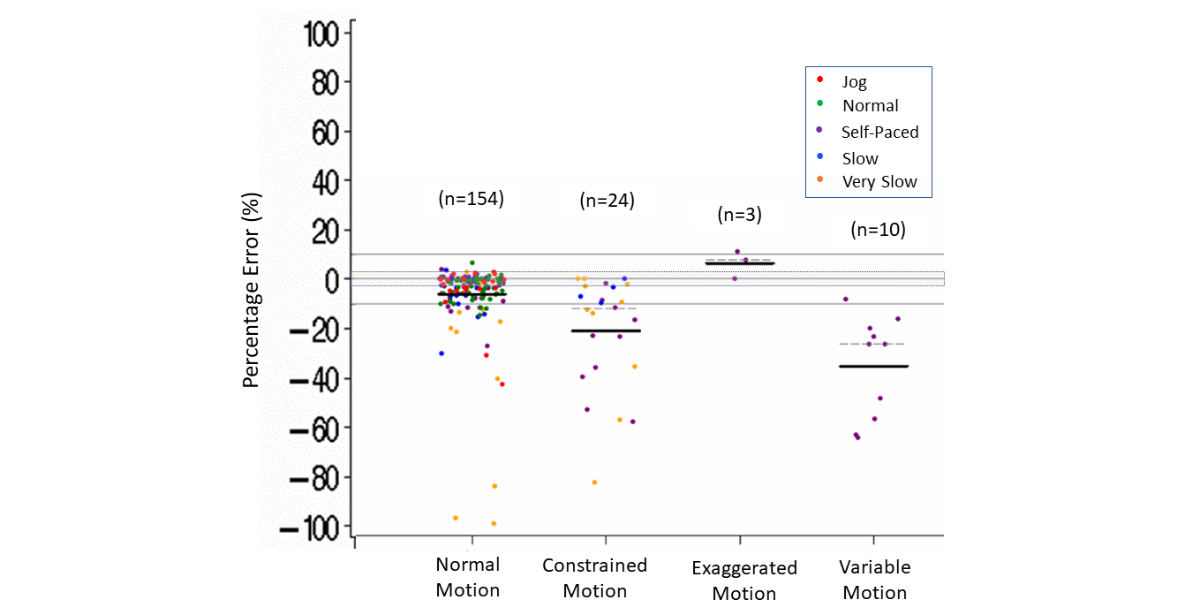
Step count percentage error in controlled settings. Body motion (normal, constrained, exaggerated, variable) by speed (jog, normal, self-paced, slow, very slow). Dark lines indicate mean (horizontal). Dashed lines indicate median (horizontal).

Within the variations of body motion during the activity, measurement error was within ±3% more than 50% (82/154) of the time during activities with normal body motion. Measurement error was below –3% more than 90% of the time for activities that involved constrained (19/24) or variable (10/10) body motion during the activity, with Fitbit tending to underestimate steps during these activities (estimated mean [median] errors varying from –35% [–26%] up to –21% [–12%]). Conversely, when the Fitbit device was worn on the wrist during exaggerated arm motion, 2 of the 3 comparisons were above 3% (Multimedia Appendix 8 and Figure 5).

We also observed that, within the different speeds of ambulation, step count accuracy appeared to be influenced further by the placement of the device on the body (Figures 3 and 4). For torso placement, measurement accuracy was within ±3% more than 60% of the time for normal (24/30), self-paced (28/44), and slow (7/11) ambulation speeds. Torso placement was lower than –3% more than 60% of the time for jogging (9/14) and more than 90% of the time for very slow (14/15) walking speeds. In addition, we observed that the underestimation of steps was largest during very slow walking when the device was worn on the torso, with 7 of these 15 comparisons having a measurement error lower than –25%. For ankle placement, 70% (11/16) of the accuracy comparisons were within ±3% measurement error for slow or very slow walking speeds. There were no accuracy comparisons for ankle placement at normal or jogging speeds and only 1 comparison for ankle placement during self-paced ambulation. For wrist placement, 90% (9/10) of time measurement error was within ±3% for jogging speeds and 75% (38/51) of the time it was lower than –3% for all other speeds.

A total of 13 studies examined Fitbit accuracy for step count in free-living conditions (20 accuracy comparisons; Multimedia Appendix 3) [36,52-63]. Of these, 8 studies were conducted in healthy young adults; 5 were conducted on older adults, of whom 3 were healthy, active older adults and 2 had mobility limitations. Duration of wear varied from 1 to 14 days. Fitbit devices were compared with ActiGraph, activPAL, or Actical accelerometers, or an Omron or Shimmer pedometer.

Across the 20 accuracy comparisons examining step count in free-living settings, 55% (n=11) were within a ±10% measurement error, 30% (n=6) were below a –10% measurement error, and 15% (n=3) were above a 10% measurement error, with a tendency for Fitbit to overestimate steps in free-living conditions relative to a research-grade criterion. When explored further, it appeared that measurement error for step count in free-living conditions varied depending on the reference criterion used, body placement of the device, and the age and mobility status of the study participants. Compared with ActiGraph or activPAL accelerometers, Fitbit step count was within a ±10% error for 6 of 8 torso comparisons and 3 of 5 wrist comparisons in healthy young adults, and in 1 comparison when worn on the torso in older adults with no mobility limitation. In 1 comparison in older adults with no mobility limitations, a Fitbit device worn on the torso overestimated steps by more than 35% relative to an Omron pedometer worn on the ankle.

In contrast, in 2 of 3 accuracy comparisons in older adults with mobility limitations, Fitbit step count error was approximately –25% lower than that of a Shimmer pedometer or an Actical accelerometer worn on the ankle when Fitbit was worn on the torso (Multimedia Appendix 9).

Our evaluation for step count accuracy in free-living settings was consistent with those of 5 other studies examining MAPE or level-of-agreement differences in daily step measures in healthy adults for Fitbit compared with an accelerometer worn on the torso or a pedometer worn on the ankle [42,64-67]. These studies reported Fitbit overestimations of median steps per day varying from 700 to 1800 steps or MAPE values greater than 10% when compared with an ActiGraph accelerometer or Omron or New Life pedometers. In contrast, 1 study showed similar measures for median steps per day for Fitbit compared with a Yamax pedometer (–55 steps/day) [67].

### Energy Expenditure

A total of 18 studies (98 accuracy comparisons) examined Fitbit device energy expenditure measurement accuracy in controlled settings compared with a reference standard of direct (2 studies) or indirect (16 studies) calorimetry (Multimedia Appendix 3) [29,34,37,39,46,68-80]. All 18 studies recruited healthy adults. Fitbit devices were worn on the torso or wrist. Of the accuracy comparisons, 88 measured energy expenditure during an activity, while 10 measured energy expenditure at rest.

**Figure 6:**
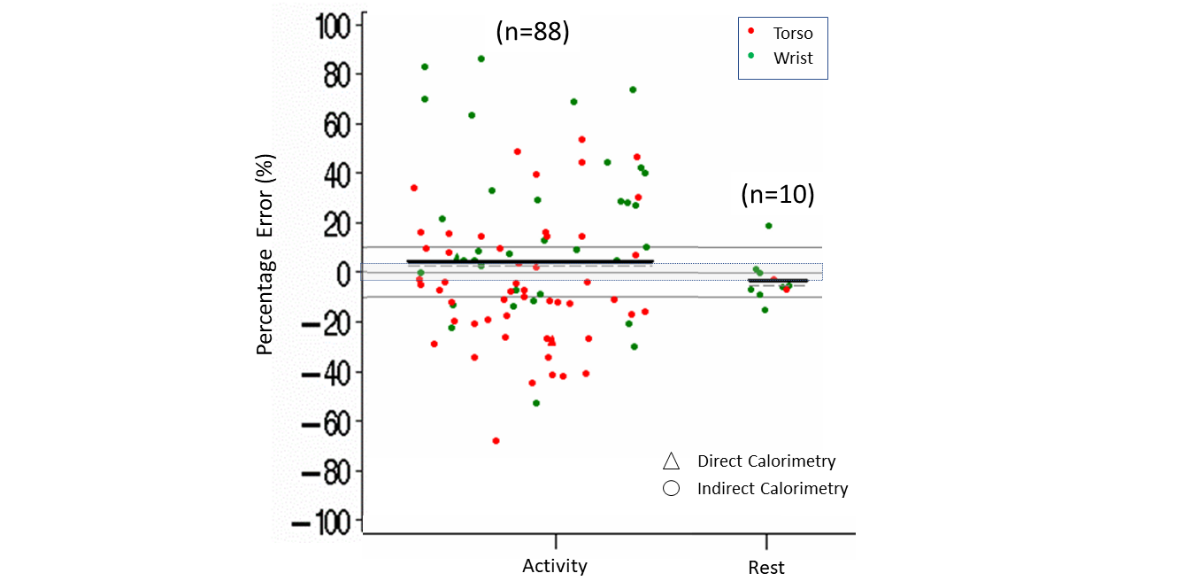
Energy expenditure percentage error in controlled settings. Activity versus rest by body placement (torso, wrist). Dark lines indicate mean (horizontal). Dashed lines indicate median (horizontal). Triangles indicate measurement by direct calorimetry. Gray shading indicates ±3% measurement error.

Findings indicated that, across the 88 activity comparisons, measurement error was rarely within ±3% (4% [n=4] within a ±3% error, 47% [n=41] below a –3% error, and 49% [n=43] above a 3% error). Overall, Fitbit showed a tendency to overestimate energy expenditure during activity (estimated mean [median] error of 4% [2%]). Across the 10 comparisons at rest, 3 were within a ±3% measurement error, with 6 lower than –3% and 1 higher than 3%, with a tendency to underestimate energy expenditure (estimated mean [median] error of –3% [–6%]) (Multimedia Appendix 8 and Figure 6).

When we explored further by factors potentially influencing energy expenditure measurement accuracy, we observed that accuracy appeared to vary with speed of ambulation, with body placement, and with variations in body motion during the activity. In addition, energy expenditure accuracy appeared to be influenced by type of ambulation. Types of ambulation included continuous ambulation on an incline or a flat surface, as well as intermittent ambulation (stop-and-start ambulation) while performing common simulated household or sporting activities (Multimedia Appendix 8).

**Figure 7:**
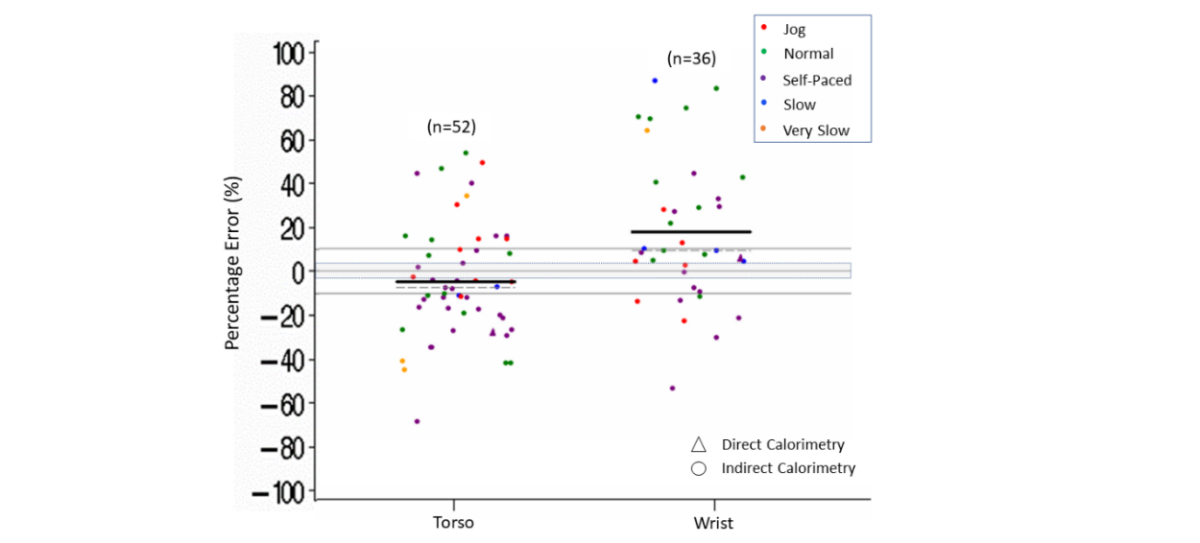
Energy expenditure percentage error in controlled settings. Body placement (torso, wrist) by speed (jog, normal, self-paced, slow, very slow). Dark lines indicate mean (horizontal). Dashed lines indicate median (horizontal). Triangles indicate measurement by direct calorimetry. Gray shading indicates ±3% measurement error.

Within the different body placements, measurement error for energy expenditure was lower than –3% more than 60% (32/52) of the time with torso placement (estimated mean [median] error of –5% [–8%]) and greater than 3% more than 60% (24/36) of the time for wrist placement (estimated mean [median] error of 18% [9%]) (Multimedia Appendix 8 and Figure 7).

**Figure 8:**
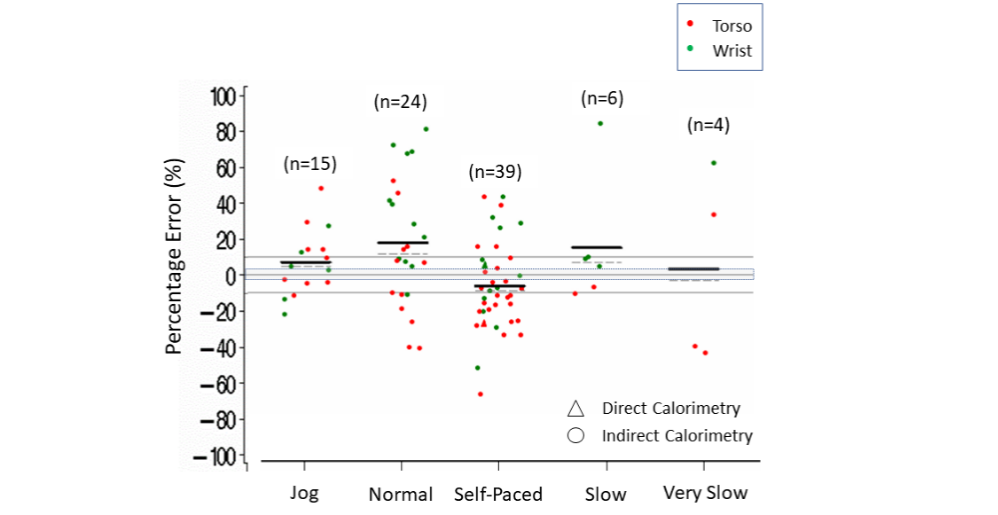
Energy expenditure percentage error in controlled settings. Speed (jog, normal, slow, self-paced, very slow) by body placement (torso, wrist). Dark lines indicate mean (horizontal). Dashed lines indicate median (horizontal). Triangles indicate measurement by direct calorimetry. Gray shading indicates ±3% measurement error.

Within the different speeds of ambulation, more than 50% of jogging (8/15) and normal (17/24) speed comparisons for energy expenditure were greater than 3% (estimated mean [median] errors varying from 7% (5%) to 18% (12%]). Conversely, more than 50% (25/39) of the self-paced ambulation comparisons were below –3% (estimated mean [median] error of –6% [–9%]). There were fewer than 10 comparisons for energy expenditure at slow and very slow ambulation speeds, with no apparent trend or pattern for measurement error noted (Multimedia Appendix 8 and Figure 8).

**Figure 9:**
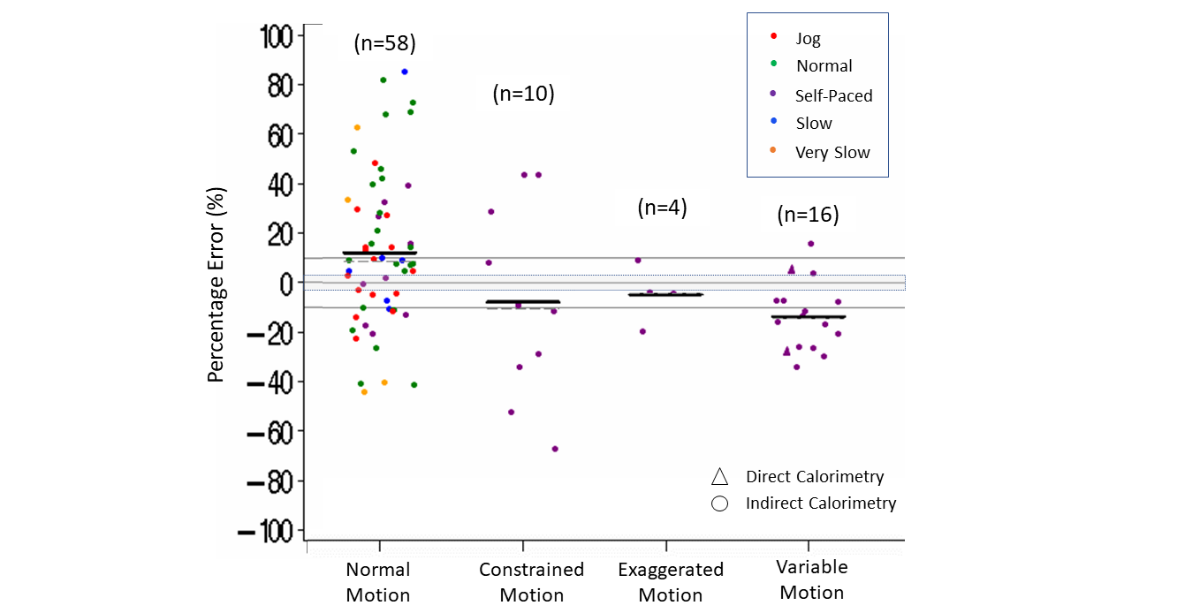
Energy expenditure percentage error in controlled settings. Motion limitations (normal, constrained, exaggerated, variable) by speed (jog, normal, self-paced, slow, very slow). Dark lines indicate mean (horizontal). Dashed lines indicate median (horizontal). Triangles indicate measurement by direct calorimetry. Gray shading indicates ±3% measurement error.

Within the different body motion parameters, more than 50% (34/58) of activities with normal body motion had an energy expenditure error greater than 3% (estimated mean [median] difference of 12% [9%]). In contrast, more than 60% of the accuracy comparisons during activities with constrained (6/10) or variable (13/16) body motion activities had an energy expenditure error lower than –3% (estimated mean [median] difference varying from –14% [–15%] to –8% [–10%]). Similarly, 3 of the 4 comparisons with exaggerated motion also had a measurement error for energy expenditure lower than –3% (Multimedia Appendix 8 and Figure 9).

**Figure 10:**
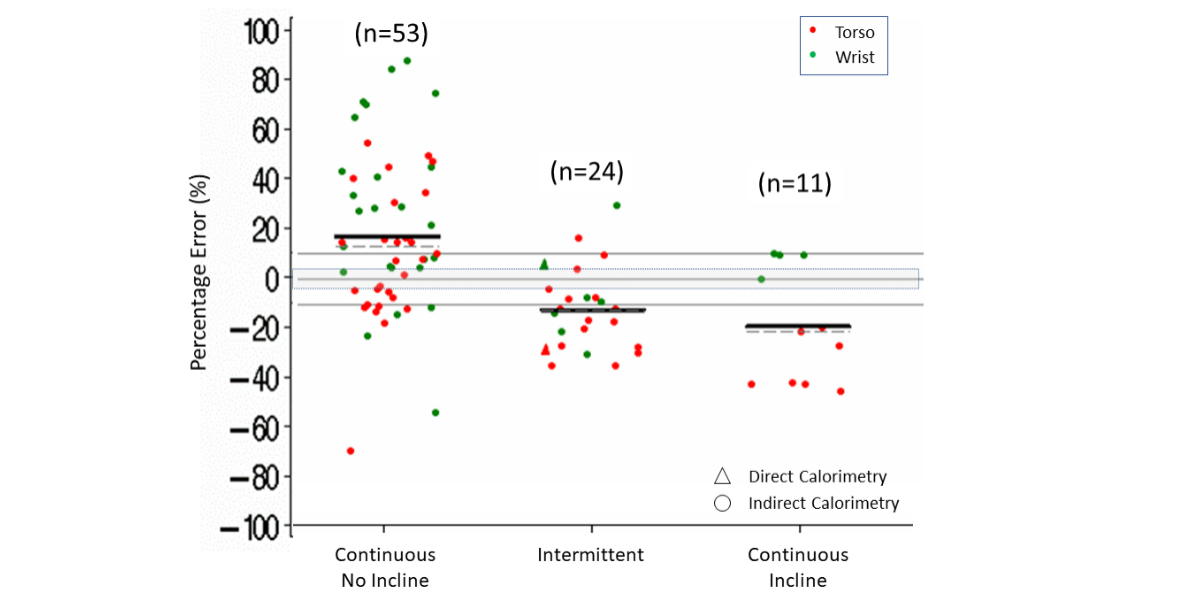
Energy expenditure percentage error in controlled settings. Type of ambulation (continuous no incline, continuous incline, intermittent) by body placement (torso, wrist). Dark lines indicate mean (horizontal). Dashed lines indicate median (horizontal). Triangles indicate measurement by direct calorimetry. Gray shading indicates ±3% measurement error.

Within the different types of ambulation, more than 60% (35/53) of the continuous ambulation activities on flat surfaces had an error for energy expenditure greater than a 3% (estimated mean [median] difference of 17% [13%]). More than 60% of the time, the error was lower than –3% with continuous ambulation activities on an incline (7/11) or intermittent ambulation during simulated household or sporting activities (18/24) (estimated mean [median] errors varying from –19% [–21%] to –12% [–12%]) (Multimedia Appendix 8 and Figure 10).

A total of 3 studies examined Fitbit device accuracy for measures of energy expenditure in healthy adults in free-living conditions compared with doubly labelled water (1 accuracy comparison) or a SenseWear accelerometer (4 accuracy comparisons; Multimedia Appendix 3) [56,77,81]. Findings from 1 study showed that Fitbit worn on the wrist tended to slightly underestimate (–7%) energy expenditure over 15 days compared with doubly labelled water [77]. All 4 accuracy comparisons with a SenseWear accelerometer were lower than a –10% measurement error (estimated mean [median] difference of –15% [–15%]) (Multimedia Appendix 9). These findings are consistent with those of 2 other accuracy studies reporting Fitbit underestimations of daily energy expenditure, with MAPE values varying from 16% to 30% for Fitbit devices compared with measurements from an ActiGraph or Actiheart accelerometer [58,70].

### Time in Activity

A total of 8 studies (28 accuracy comparisons) examined Fitbit device measures in free-living settings for time spent in different intensities of activity compared with measures from an ActiGraph accelerometer worn on the torso or Actical accelerometer worn on the ankle (Multimedia Appendix 3) [52,53,56,57,59-62]. Of these studies, 5 were conducted on healthy young adults and 3 were conducted on older adults living with a variety of chronic diseases. The duration of wear varied from 2 to 9 days. Studies examined time spent in sedentary, light, moderate, vigorous, or moderate to vigorous physical activity during waking hours.

Notably, the Fitbit device and the reference criterion accelerometers across the studies used variable cutoff points for defining intensity levels of physical activity. Despite these differences, 3 of the 4 accuracy comparisons for sedentary time had an error lower than –10% when compared with ActiGraph (torso) or Actical (ankle) accelerometers. Compared with Actical (ankle) or ActiGraph (torso) accelerometers, more than 80% (21/24) of accuracy comparisons for time spent in light to vigorous activity time had a measurement error greater than 10% (estimated mean [median] difference varying from 44% [52%] to 632% [390%]) (Multimedia Appendix 9).

Our observation of marked overestimation of time spent in higher-intensity activity was consistent with those of 2 other studies reporting Fitbit overestimations of moderate to vigorous physical activity in free-living settings compared with an ActiGraph accelerometer (MAPEs >30%) [58,66]. In contrast to our finding of Fitbit underestimation of sedentary time during the day, 1 study reported Fitbit overestimation of combined night (sleep) and daytime sedentary time (MAPE ~10%) when compared with an activPAL accelerometer worn on the thigh. [66].

### Sleep

A total of 3 studies examined sleep in controlled settings (12 accuracy comparisons), comparing a Fitbit worn on the wrist against reference-standard polysomnography over 1 night of sleeping in a laboratory (Multimedia Appendix 3) [82-84]. All 3 studies included young adults, 2 comprising healthy participants and 1 comprising individuals living with depression. All 3 studies examined measures of sleep in a normal-mode setting, and all reported Fitbit overestimation of total sleep time and sleep efficiency by more than 10%. On the other hand, 1 study examined total sleep time and sleep efficiency in the sensitive sleep mode, reporting Fitbit underestimation of both by more than 15% [82]. One study also examined sleep-onset latency (minutes to initial sleep) and time awake after sleep onset in normal and sensitive sleep modes and reported measurement errors varying from 12% to 180%, with an opposite tendency for either over- or underestimations of these sleep parameters depending on the sleep-mode setting (Multimedia Appendix 8).

A total of 3 studies (5 accuracy comparisons) reported sleep measurement accuracy in healthy young adults in free-living settings comparing a Fitbit device worn on the wrist with a SenseWear or Actiwatch accelerometer also worn on the wrist (Multimedia Appendix 3) [56,81,85]. Duration of wear varied from 1 to 13 nights of home sleep. There were 4 comparisons for measures of time in bed, with all 4 reporting measurement errors within ±10% compared with a SenseWear accelerometer. One study also reported very similar measures for time in bed (–0.4%) by a Fitbit device compared with an Actiwatch accelerometer. One study also reported a slight overestimate (6%) of sleeping minutes for Fitbit compared with an Actiwatch accelerometer [85] (Multimedia Appendix 9). These findings are consistent with those of 2 other studies reporting Fitbit overestimations of sleeping time compared with a portable sleep monitor (MAPE approximately 10%) or Actiwatch accelerometer (approximately 10 minutes per night) [66,86].

### Distance

There were 2 studies (17 accuracy comparisons) examining Fitbit device distance measurement accuracy in a controlled setting in healthy young adults (Multimedia Appendix 3) [33,48]. Both studies reported a Fitbit tendency to overestimate distance at slower and self-paced ambulation speeds (varying from 5% [torso] to 25% [wrist]) and underestimate distance at brisk walking or jogging speeds (varying from –15% [wrist] to –5% [torso]). During normal speed ambulation, torso placement tended to overestimate distance (10%), and wrist placement tended to slightly underestimate distance (–3%). These findings are consistent with 1 additional study reporting Fitbit overestimations of distances at slower walking speeds varying from 5% to 15% and underestimations of distance by more than 10% during running activities (Multimedia Appendix 8) [87].

## Discussion

### Principal Findings

This review adds to the existing literature, as it is the first, to our knowledge, to systematically examine and report Fitbit device measurement accuracy in controlled and free-living settings for measures of step count, energy expenditure, sleep, time in activity, and distance in healthy adults or adults living with any health condition or disability.

Findings across many studies suggested that, approximately 50% of the time, Fitbit devices were likely to provide accurate measures (within ±3%) of steps in controlled testing conditions, with an overall tendency to underestimate steps. Findings also indicated that step count accuracy was likely to improve if the device was worn on the torso during normal or self-paced walking activities, worn on the wrist during jogging activities, and worn on the ankle during slow or very slow walking activities. Findings from several studies examining step count in free-living settings also showed that, approximately 50% of the time, Fitbit devices were likely to provide relatively accurate (within ±10%) measures of steps compared with research-grade accelerometers or pedometers when worn on the torso or wrist in healthy adults with no mobility limitations, with a tendency to overestimate steps in free-living settings.

Consistent findings across studies in controlled-testing settings indicated that Fitbit devices were also more likely to provide notable underestimations of step count during activities with very slow ambulation, particularly when worn on the torso, where body motion may be constrained by mobility limitations or walking while pushing a walker or stroller, and during activities that simulate household or sporting activities that involve stop-and-start ambulation throughout the task. Findings from a few studies in free-living conditions suggested that, compared with a research-grade accelerometer or pedometer worn at the ankle, a Fitbit device worn on the torso may markedly overestimate steps in older adults with no mobility limitation and markedly underestimate steps in older adults with limited mobility.

There were also consistent findings from many studies examining energy expenditure in controlled settings, indicating that Fitbit devices were rarely likely to provide accurate measures of energy expenditure. Findings suggested that Fitbit was more likely to markedly overestimate energy expenditure when worn on the wrist and when walking at normal adult walking speeds on flat surfaces. On the contrary, Fitbit was more likely to underestimate energy expenditure when worn on the torso, with a tendency to markedly underestimate energy expenditure during inclined ambulation, during activities with constrained or variable body motion throughout the activity, and during simulated household or sporting activities that involve stop-and-start ambulation. Findings from 1 study for measures of energy expenditure in free-living settings suggested that Fitbit and doubly labelled water may provide similar measures of total energy expenditure over a 2-week period. However, findings from a few studies in free-living settings suggested that Fitbit devices may provide notable underestimations of daily energy expenditure compared with a SenseWear accelerometer.

A few studies examined Fitbit measurement accuracy for time spent in different intensity of activity in free-living settings. Across these studies, there was consistent evidence to suggest that, compared with research-grade accelerometers, Fitbit devices may underestimate sedentary time and progressively overestimate time in spent in activity as intensity of activity increases. Similarly, a few studies examined the accuracy for measures of sleep in controlled or free-living settings. Consistent evidence from these studies suggested that Fitbit may not provide accurate measures of sleep quality or quantity in a controlled-testing setting compared with polysomnography. However, there was some indication that Fitbit may provide relatively similar measures to SenseWear or Actiwatch accelerometers for time spent in bed and time sleeping in free-living settings. Finally, findings from 2 studies suggested that Fitbit may overestimate distance with slower walking speeds and progressively underestimate distance as walking speed increases.

Most of the studies included in this review were published in the last 2 years, with studies primarily examining measurement accuracy for models of Fitbit activity trackers introduced prior to 2015. The included studies mainly focused on step count and energy expenditure outcome measurement accuracy, with only a few of the studies examining measurement accuracy for sleep, distance, or time in activity. As well, the vast majority of studies included only healthy participants, with few including older adults, and fewer still including any adult living with disease or functional limitation. Overall, the quality of the included studies was excellent in terms of study design, reporting of missing data, and use of acceptable accuracy evaluations. However, some studies did not clearly identify how they may have handled missing data in their analyses, and few comprised more than 50 participants.

Most of the studies focused on measurement accuracy in controlled-testing environments comparing measurements from a Fitbit device against a reference-standard criterion. Standardized and controlled testing environments allow for evaluations of “true” measurement accuracy but do not necessarily reflect device measurement accuracy in uncontrolled or free-living settings, which are the intended environments for Fitbit activity tracker use. However, it is very difficult to measure true device accuracy in free-living conditions, as the reference-standard criterion measures generally cannot be used over a number of days while someone is conducting their usual daily activities. Therefore, the studies examining Fitbit device measurement accuracy in free-living conditions examined the accuracy of Fitbit device measures relative to an established research-grade criterion device measure of the same outcome when worn at the same time in free-living conditions.

For the purposes of this review, we defined satisfactory levels of measurement accuracy based on previously published standards for acceptable accuracy of step count in controlled (±3%) and free-living (±10%) settings [19-22]. Given that we were not able to identify published standards for accuracy of other outcome measures, we applied these same cutoff points for acceptable limits of measurement accuracy for all outcomes. However, we provide details of our descriptive analyses in the supplemental summary tables and offer visual representations for error estimations in the figures to allow for independent assessment of alternative definitions for acceptable limits for measurement accuracy by Fitbit devices.

### Limitations

Our review has some potential limitations. These include the decision to include only data that were published in peer-reviewed journals and to exclude non-English studies. These decisions may have introduced a level of bias in our analyses and interpretation. In addition, we included all studies, independent of potential risk of bias. Moreover, the descriptive analyses and subsequent point estimations for percentage measurement error (ie, potential bias) gave equal weighting to accuracy comparisons with different sample sizes and variations in significance levels, which may misrepresent the true point estimate for measurement error for some of the testing conditions examined in this review [17,18]. Allowing for these potential limitations, and the limited number of studies examining Fitbit measurement accuracy for sleep, distance, and time spent in activity, we note that discretion should be exercised when considering our evaluations of the potential accuracy for these outcome domains. To address this gap in the literature, further high-quality research examining Fitbit measurement accuracy for sleep, time in activity, and distance is warranted.

We should identify as well that defining relative (in)accuracy of a Fitbit device in free-living settings does not define true measurement (in)accuracy, as neither the Fitbit device nor the reference device was compared to a reference-standard criterion. Rather, relative inaccuracy of a Fitbit defines only the likelihood that a Fitbit device will provide different values for measures of the same outcome when compared with a research-grade criterion in free-living conditions.

It is also important to clarify that we derived estimates of Fitbit device measurement accuracy in this review from studies that used different models of Fitbit, which might have different versions of firmware, software, and data processing algorithms. Since the design details for the devices and software are proprietary information, we were not able to determine whether and what modifications have been made by the company over time. Nonetheless, we indirectly explored the potential effect of differences in model design over time by using body placement for the device as a proxy, as the earlier models (eg, Classic, One, Zip, and Ultra) were worn on the torso, whereas the later models (eg, Flex, Charge, and Surge) were worn on the wrist. Therefore, some of the variability in error estimations with different body placement may be related in part to differences in device design or in analysis protocols over time.

Finally, our finding of potential limitations in Fitbit device measurement accuracy in a variety of testing conditions does not imply that Fitbit device measurement accuracy will remain static. Rather, it is very likely that accuracy will improve as technological advances in the firmware are implemented. As well, given the ability for Fitbit to tap into metadata from millions of users worldwide and apply advanced algorithms to better identify complex patterns of motion, it is likely that evolving software upgrades will also lead to improved measurement accuracy. Furthermore, our findings do not negate the value of using Fitbit activity trackers in the manner for which the devices were intended, which is for self-monitoring of physical activity patterns and motivating individuals to achieve their physical activity goals [88-91].

### Conclusion

Fitbit devices are most likely to provide accurate measures of steps in adults with no mobility limitations, when the device is worn on the torso while walking at normal or self-paced walking speeds. However, Fitbit devices are unlikely to provide accurate measures of energy expenditure. Limited evidence suggests that Fitbit activity trackers may not provide accurate measures for sleep, distance, or time spent in activity; however, further accuracy studies are warranted.

### Implications

Other than for measures of steps in adults with no limitations in mobility, discretion should be used when considering the use of Fitbit devices as an outcome measurement tool in research or to inform health care decisions, as there are seemingly a limited number of situations where the device is likely to provide accurate measurement.

## Appendix

Multimedia Appendix 1 Search strategy example.

Multimedia Appendix 2 Data extraction framework.

Multimedia Appendix 3 Master data – percentage error values (all accuracy comparisons).

Multimedia Appendix 4 Modified Consensus-Based Standards for the Selection of Health Status Measurement Instruments (COSMIN) criteria.

Multimedia Appendix 5 Data coding (steps and energy expenditure in controlled settings).

Multimedia Appendix 6 Study characteristics.

Multimedia Appendix 7 Accuracy evaluations reported and risk-of-bias assessment.

Multimedia Appendix 8 Controlled settings: accuracy – measurement error.

Multimedia Appendix 9 Free-living settings: relative accuracy – measurement error.

## Abbreviations

COSMIN: Consensus-Based Standards for the Selection of Health Status Measurement Instruments
MAPE: mean or median absolute percentage error
