# Supplementary material for: Accuracy of Fitbit Devices: Systematic Review and Narrative Syntheses of Quantitative Data
Source: JMIR Mhealth Uhealth. 2018 Aug 9;6(8):e10527. doi: 10.2196/10527 (PMC6107736; doi:10.2196/10527)
Supplement: Multimedia Appendix 1 [file mhealth_v6i8e10527_app1.pdf]

**Search Strategy Example:** PubMedEmbase (via OvidSP, 2011 to 2017; last reviewed December 15, 2017).	
1	Fitbit*.mp.[mp=ti,ab,hw,tn,ot,dm,mf,dv,kw,fx,nm,kf,px,rx,an,eu,pm,ui,sy]
2	Limit 1 to yr="2011 - 2017"
3	accura*.mp.[mp=ti,ab,hw,tn,ot,dm,mf,dv,kw,fx,nm,kf,px,rx,an,eu,pm,ui,sy]
4	Limit 3 to yr="2011 - 2017"
5	valid*.mp.[mp=ti,ab,hw,tn,ot,dm,mf,dv,kw,fx,nm,kf,px,rx,an,eu,pm,ui,sy]
6	Limit 5 to yr="2011 - 2017"
7	compar*.mp.[mp=ti,ab,hw,tn,ot,dm,mf,dv,kw,fx,nm,kf,px,rx,an,eu,pm,ui,sy]
8	Limit 7 to yr="2011 - 2017"
9	relation*.mp.[mp=ti,ab,hw,tn,ot,dm,mf,dv,kw,fx,nm,kf,px,rx,an,eu,pm,ui,sy]
10	Limit 9 to yr="2011 - 2017"
11	6 or 7 or 9
12	associa*.mp.[mp=ti,ab,hw,tn,ot,dm,mf,dv,kw,fx,nm,kf,px,rx,an,eu,pm,ui,sy]
13	Limit 12 to yr="2011 - 2017"
14	equival*.mp.[mp=ti,ab,hw,tn,ot,dm,mf,dv,kw,fx,nm,kf,px,rx,an,eu,pm,ui,sy]
15	Limit 14 to yr="2011 - 2017"
16	Agreement.mp.[mp=ti,ab,hw,tn,ot,dm,mf,dv,kw,fx,nm,kf,px,rx,an,eu,pm,ui,sy]
17	Limit 16 to yr="2011 - 2017"
18	2 and 4
19	2 and 11
20	2 and 17
