# Supplementary material for: Accuracy of Fitbit Devices: Systematic Review and Narrative Syntheses of Quantitative Data
Source: JMIR Mhealth Uhealth. 2018 Aug 9;6(8):e10527. doi: 10.2196/10527 (PMC6107736; doi:10.2196/10527)
Supplement: Multimedia Appendix 2 [file mhealth_v6i8e10527_app2.pdf]

**Data Extraction Framework**

Study Characteristics:

- Study [first author (last name), year (month), country, publication type (article, short report, letter to editor)].
- Participants [sample size (#), age (years), gender (# M/F), health / functional status (healthy, disease(s), functional / mobility limitation(s))].

Accuracy Comparison(s) Within Each Study:

- Setting(s) [controlled, free-living].
- FitBit device characteristics [model(s), body placement(s)].
- Outcome measure(s) examined [steps, energy expenditure (EE), time in activity, distance, sleep].
- Acceptable gold standard (i.e., controlled setting) criterion (type, details) [Steps: visual observation / count; *EE:* indirect / direct calorimetry; Sleep: polysomnography; Distance: measurement].
- Acceptable research standard (i.e., free living setting) criterion (type, details) [Steps: accelerometer / pedometer; EE: doubly labelled water / accelerometer; Sleep: portable monitor / accelerometer; Distance: GPS; Time in Activity: accelerometer].
- Acceptable accuracy analyses (Fitbit vs criterion): [Group mean or percent difference (values or %), Median / Mean Absolute Percent Error – MAPE (%), Level of Agreement (values / direction)]

NOTE: Data must be reported in / extractable from the published article.
