# Supplementary material for: Accuracy of Fitbit Devices: Systematic Review and Narrative Syntheses of Quantitative Data
Source: JMIR Mhealth Uhealth. 2018 Aug 9;6(8):e10527. doi: 10.2196/10527 (PMC6107736; doi:10.2196/10527)
Supplement: Multimedia Appendix 3 [file mhealth_v6i8e10527_app3.pdf]

Author (Year)	Model	Location	Activity Setting	Outcome Measure	Criterion tool (Model)	Unit of measurement	Activity Type	Activity Details	% Group Difference
Adam Noah (2013)	Classic (x2)	Hip	Controlled	Energy Expenditure	Indirect calorimetry (K4b2 Cosmed)	kcal/min	Walk:Treadmill	Walk (5.63 kph / 3.5 mph) [1.56 m/s]	-10.02%
Adam Noah (2013)	Ultra (x2)	Hip	Controlled	Energy Expenditure	Indirect calorimetry (K4b2 Cosmed)	kcal/min	Walk:Treadmill	Walk-(5.63 kph) [1.56 m/s]	-11.04%
Adam Noah (2013)	Classic (x2)	Hip	Controlled	Energy Expenditure	Indirect calorimetry (K4b2 Cosmed)	kcal/min	Walk Incline:Treadmill	Walk-5% Incline (5.63 kph / 3.5 mph) [1.56 m/s]	-41.42%
Adam Noah (2013)	Ultra (x2)	Hip	Controlled	Energy Expenditure	Indirect calorimetry (K4b2 Cosmed)	kcal/min	Walk Incline:Treadmill	Walk-5% Incline (5.63 kph 3.5 mph) [1.56 m/s]	-41.08%
Adam Noah (2013)	Classic (x2)	Hip	Controlled	Energy Expenditure	Indirect calorimetry (K4b2 Cosmed)	kcal/min	Jog: Treadmill	Jog (8.85 kph / 5.5mph) [2.46 m/s]	-11.46%
Adam Noah (2013)	Ultra (x2)	Hip	Controlled	Energy Expenditure	Indirect calorimetry (K4b2 Cosmed)	kcal/min	Jog: Treadmill	Jog (8.85 kph / 5.5 mph) [2.46 m/s]	9.80%
Adam Noah (2013)	Classic (x2)	Hip	Controlled	Energy Expenditure	Indirect calorimetry (K4b2 Cosmed)	kcal/min	Stairs	Stair Stepping (30.5 cm hieght at 96 steps / min),0.48 m/s	-40.51%
Adam Noah (2013)	Ultra (x2)	Hip	Controlled	Energy Expenditure	Indirect calorimetry (K4b2 Cosmed)	kcal/min	Stairs	Stair Stepping (30.5 cm hieght at 96 steps / min), 0.48 m/s	-44.28%
Alharbi (2016)	Flex	Wrist	Freeliving	Steps	Accelerometry (Actigraph GT3X)	Steps / day	Normal Daility Activities	4 days (2 weekday, 2 weekend), waking hours	13.0%
Alharbi 2016	Flex	Wrist	Freeliving	Time in Activity	Accelerometry (Actigraph GT3X)	MVPA (Mins / Day)	4 days (2 weekday, 2 weekend), [illegible]	MVPA: > = 2020 counts / minute. FB: > 3 METS	10%
Bai (2016)	Flex	Wrist	Controlled	Energy Expenditure	Indirect calorimetry (Oxycon Mobile)	Total EE (kcal / 20-min)	Resting	Sedentary Behaviors	-15.07%
Bai (2016)	Flex	Wrist	Controlled	Energy Expenditure	Indirect calorimetry (Oxycon Mobile)	Total EE (kcal / 25-min)	Ambulation: Treadmill	Aerobic Activities:	26.80%
Bai (2016)	Flex	Wrist	Controlled	Energy Expenditure	Indirect calorimetry (Oxycon Mobile)	Total EE (kcal / 25-min)	Physical Activity: Resistance	Resistance Activities	-29.83%
Balto (2016)	One	Hip	Controlled	Steps	Visual Observation / Count	Number: Steps / 500 steps	Walk: Treadmill	500 steps, comfortable walking speed	-13.07%
Balto (2016)	Flex	Wrist	Controlled	Steps	Visual Observation / Count	Number: Steps / 500 steps	Walk: Treadmill	500 steps comfortable walking speed	-1.72%
Battenberg (2017)	One	Waist	Controlled	Steps	Visual Observation / Count	Distance: steps/1200 m	Walking: Hallway	steps/ 3 x 400 m (brisk walking, self-paced)	0.29%
Battenberg (2017)	One	Waist	Controlled	Steps	Visual Observation / Count	Distance: steps/1200 m	Walking: Hallway	steps/3 x 400 m (run, self-paced)	-0.07%
Battenberg (2017)	One	Waist	Controlled	Steps	Visual Observation / Count	Distance: steps/ 30m	Walking: Hallway	walk 3 x 10 m (self-paced, household, slow)	-0.36%
Battenberg (2017)	One	Waist	Controlled	Steps	Visual Observation / Count	Number: # steps/10	Stairs: Ascend	steps/10 steps (stairs, ascend)	3.67%
Battenberg (2017)	One	Waist	Controlled	Steps	Visual Observation / Count	Number: # steps/10	Stairs: Descend	steps/10 steps (stairs, descend)	-0.78%
Battenberg (2017)	Force	Wrist	Controlled	Steps	Visual Observation / Count	Distance: steps/1200 m	Walking: Hallway	steps/ 3 x 400 m (brisk walking, self-paced)	-7.90%
Battenberg (2017)	Force	Wrist	Controlled	Steps	Visual Observation / Count	Distance: steps/1200 m	Walking: Hallway	steps/3 x 400 m (run, self-paced)	-2.92%
Battenberg (2017)	Force	Wrist	Controlled	Steps	Visual Observation / Count	Distance: steps/30m	Walking: Hallway	walk 3 x 10 m (self-paced, household, slow)	-11.50%
Battenberg (2017)	Force	Wrist	Controlled	Steps	Visual Observation / Count	Number: # steps/10	Stairs: Ascend	steps/10 steps (stairs, ascend)	0.78%
Battenberg (2017)	Force	Wrist	Controlled	Steps	Visual Observation / Count	Number: # steps/10	Stairs: Descend	steps/10 steps (stairs, descend)	-6.33%
Battenberg (2017)	One	Waist	Controlled	Steps	Visual Observation / Count	Time: Steps/2 min	Walking: Treadmill	steps/2 min, 1.0 mph, 0.45 m/s	-19.80%
Battenberg (2017)	One	Waist	Controlled	Steps	Visual Observation / Count	Time: Steps/2 min	Walking: Treadmill	steps/2 min, 1.5 mph, 0.67 m/s	-1.72%
Battenberg (2017)	One	Waist	Controlled	Steps	Visual Observation / Count	Time: Steps/2 min	Walking: Treadmill	steps/2 min, 1.8 mph, 0.80 m/s	0.27%
Battenberg (2017)	One	Waist	Controlled	Steps	Visual Observation / Count	Time: Steps/2 min	Walking: Treadmill	steps/2 min, 2.0 mph, 0.89 m/s	-0.04%
Beevi (2015)	Zip	Hip	Controlled	Steps	Visual Observation / Count	Number: Steps / 100 steps	Walk: Treadmil	One hundred steps @ 3 km / hour) x4 [0.83 m/s]	0.70%
Beevi (2015)	Zip	Hip	Controlled	Steps	Visual Observation / Count	Number: Steps / 100 steps	Walk: Treadmil	One hundred steps @ 1 km / hour x4 [0.28 m/s]	-95.98%
Beevi (2015)	Zip	Hip	Controlled	Steps	Visual Observation / Count	Number: Steps / 100 steps	Walk: Treadmil	One hundred steps @ 2 km / hour) x4 [0.56 m/s]	-17.27%
Brewer (2017)	Charge HR,	Wrist	Freeliving	Steps	Accelerometry (Actigraph GT3X))	Steps / day	Normal Daility Activities	7 days, waking hours	30%
Brewer (2017)	Charge HR, Charge [illegible]	Wrist	Freeliving	Time in Activity	Accelerometry (Actigraph GT3X))	MVPA (Mins / Day)	7 days, waking hours	AG not defined, FB: active minutes	3%
Brooke (2016)	Flex	Wrist	Free Living	Sleep	Accelerometry (SW Mini)	Total Bed Time (min)	Home sleep	1 night	9%
Brooke (2016)	Charge HR	Wrist	Free Living	Sleep	Accelerometry (SW Mini)	Total Bed Time (min)	Home sleep	1 night	4%
Brooke (2016)	Flex	Wrist	Free Living	Energy Expenditure	Accelerometry (SenseWear model MF)	Total EE (kcal / day)	Normal Daily Activities	24 h	-14.9%
Brooke (2016)	Charge HR	Wrist	Free Living	Energy Expenditure	Accelerometry (SenseWear model MF)	Total EE (kcal / day)	Normal Daily Activities	24 h	-13.7%
Chen 2016	Flex	Wrist	Controlled	Steps	Visual Observation / Count	Time: Steps / 5 min	Walk: Push Stroller	pushing a stroller 5 min (dominant)	-52.50%
Chen 2016	Flex	Wrist	Controlled	Steps	Visual Observation / Count	Time: Steps / 5 min	Walk: Push Stroller	pushing a stroller,5 min (non-dominant)	-35.60%
Chen 2016	Flex	Wrist	Controlled	Steps	Visual Observation / Count	Time: Steps / 5 min	Walk: Carry Bag	carrying a bag, 5 min (non-dominant)	7.50%
Chen 2016	Flex	Wrist	Controlled	Steps	Visual Observation / Count	Time: Steps / 5 min	Walk: Carry Bag	carrying a bag, 5 min (dominant)	11.10%
Chen 2016	Flex	Wrist	Controlled	Steps	Visual Observation / Count	Time: Steps / 5 min	Stair: Down	downstairs, 5 min (non-dominant)	-9.00%
Chen 2016	Flex	Wrist	Controlled	Steps	Visual Observation / Count	Time: Steps / 5 min	Stair: Up	upstairs, 5 min (non-dominant)	-7.80%
Chen 2016	Flex	Wrist	Controlled	Steps	Visual Observation / Count	Time: Steps / 5 min	Stair: Up	upstairs, 5 min (dominant)	-6.50%
Chen 2016	Flex	Wrist	Controlled	Steps	Visual Observation / Count	Time: Steps / 5 min	Stair: Down	downstairs, 5 min (dominant)	-6.00%
Chen 2016	Flex	Wrist	Controlled	Steps	Visual Observation / Count	Time: Steps / 5 min	Jog: Treadmill	5 min, jogging = 134 m/min 2.2 m/s (dominant)	-2.10%
Chen 2016	Flex	Wrist	Controlled	Steps	Visual Observation / Count	Time: Steps / 5 min	Jog: Treadmill	5 min, jogging = 134 m/min 2.2 m/s (non-dominant)	2.70%

Chen 2016	Flex	Wrist	Controlled	Steps	Visual Observation / Count	Time: Steps / 5 min	Walk: treadmill	5 min, moderate walk = 80 m/min, 1.3 m/s (non-dominant)	-7.50%
Chen 2016	Flex	Wrist	Controlled	Steps	Visual Observation / Count	Time: Steps / 5 min	Walk: treadmill	5 min fast walk = 107 m / min (1.78 m/s) (non-dominant)	-6.20%
Chen 2016	Flex	Wrist	Controlled	Steps	Visual Observation / Count	Time: Steps / 5 min	Walk: treadmill	5 min, moderate walk = 80 m/min, 1.3 m/s (dominant)	-5.90%
Chen 2016	Flex	Wrist	Controlled	Steps	Visual Observation / Count	Time: Steps / 5 min	Walk: treadmill	5 min fast walk = 107 m / min (1.78 m/s) (dominant)	-4.90%
Chen 2016	Flex	Wrist	Controlled	Steps	Visual Observation / Count	Time: Steps / 5 min	Walk: treadmill	5 min, slow walk= 54m/min (0.9 m/s) (dominant)	-10.10%
Chen 2016	Flex	Wrist	Controlled	Steps	Visual Observation / Count	Time: Steps / 5 min	Walk: treadmill	5 min, slow walk= 54m/min 0.9 m/s (non -dominant)	-6.60%
Chow (2017)	One	Waist	Controlled	Steps	Visual Observation / Count	Time: steps/3 min	Walking: Treadmill	steps/3 min 5 km/h 1.39 m/s	0.30%
Chow (2017)	One	Waist	Controlled	Steps	Visual Observation / Count	Time: steps/3 min	Walking: Treadmill	steps/3 min 6.5 km/h 1.8 m/s	1.20%
Chow (2017)	One	Waist	Controlled	Steps	Visual Observation / Count	Time: steps/3 min	Jog: Treadmill	steps/3 min 8 km/h 2.2 m/s	-0.30%
Chow (2017)	One	Waist	Controlled	Steps	Visual Observation / Count	Time: steps/3 min	Jog: Treadmill	steps/3 min 10 km/h 2.8 m/s	-0.40%
Chow (2017)	One	Waist	Controlled	Steps	Visual Observation / Count	Time: steps/3 min	Jog: Treadmill	steps/3 min 12 km/h 3.3 m/s	-1.10%
Chow (2017)	Charge	Wrist	Controlled	Steps	Visual Observation / Count	Time: steps/3 min	Walking: Treadmill	steps/3 min 5 km/h 1.39 m/s	-7.90%
Chow (2017)	Charge	Wrist	Controlled	Steps	Visual Observation / Count	Time: steps/3 min	Walking: Treadmill	steps/3 min 6.5 km/h 1.8 m/s	-10.10%
Chow (2017)	Charge	Wrist	Controlled	Steps	Visual Observation / Count	Time: steps/3 min	Jog: Treadmill	steps/3 min 8 km/h 2.2 m/s	-3.60%
Chow (2017)	Charge	Wrist	Controlled	Steps	Visual Observation / Count	Time: steps/3 min	Jog: Treadmill	steps/3 min 10 km/h 2.8 m/s	0.30%
Chow (2017)	Charge	Wrist	Controlled	Steps	Visual Observation / Count	Time: steps/3 min	Jog: Treadmill	steps/3 min 12 km/h 3.3 m/s	0.00%
Chow (2017)	Flex	Wrist	Controlled	Steps	Visual Observation / Count	Time: steps/3 min	Walking: Treadmill	steps/3 min 5 km/h 1.39 m/s	-8.60%
Chow (2017)	Flex	Wrist	Controlled	Steps	Visual Observation / Count	Time: steps/3 min	Walking: Treadmill	steps/3 min 6.5 km/h 1.8 m/s	-11.90%
Chow (2017)	Flex	Wrist	Controlled	Steps	Visual Observation / Count	Time: steps/3 min	Jog: Treadmill	steps/3 min 8 km/h 2.2 m/s	-3.30%
Chow (2017)	Flex	Wrist	Controlled	Steps	Visual Observation / Count	Time: steps/3 min	Jog: Treadmill	steps/3 min 10 km/h 2.8 m/s	1.80%
Chow (2017)	Flex	Wrist	Controlled	Steps	Visual Observation / Count	Time: steps/3 min	Jog: Treadmill	steps/3 min 12 km/h 3.3 m/s	-1.50%
Chowdhury (2017)	Charge HR	Wrist	Controlled	Energy Expenditure	Indirect calorimetry (COSMED K4b)	kcal/min	ADL, Typing	5 minutes, Seated typing on laptol	-5.96%
Chowdhury (2017)	Charge HR	Wrist	Controlled	Energy Expenditure	Indirect calorimetry (COSMED K4b)	kcal/min	ADL, Dishwasher	5 minutes, Loading and unloading	-8.94%
Chowdhury (2017)	Charge HR	Wrist	Controlled	Energy Expenditure	Indirect calorimetry (COSMED K4b)	kcal/min	ADL, Sweeping	5 minutes, Light objects across 3 m distance	29.03%
Chowdhury (2017)	Charge HR	Wrist	Controlled	Energy Expenditure	Indirect calorimetry (COSMED K4b)	kcal/min	ADL, Stairs	5 minutes, Ascending and decending one flight	-0.28%
Chowdhury (2017)	Charge HR	Wrist	Controlled	Energy Expenditure	Indirect calorimetry (COSMED K4b)	kcal/min	Walk: Treadmill	10 minutes, 1-1.3 m/s [4 or 4.8 km/h]	69.66%
Chowdhury (2017)	Charge HR	Wrist	Controlled	Energy Expenditure	Indirect calorimetry (COSMED K4b)	kcal/min	Loaded Walk - Carrying Two [illegible]	10 minutes, 6 kg for females, 10 kg for males, shopping bags	43.99%
Chowdhury (2017)	Charge HR	Wrist	Controlled	Energy Expenditure	Indirect calorimetry (COSMED K4b)	kcal/min	Cycle (stationary)	10 minutes, 75 W for females, 100 W for males	-52.55%
Chowdhury (2017)	Charge HR	Wrist	Controlled	Energy Expenditure	Indirect calorimetry (COSMED K4b)	kcal/min	Run - Treadmill	10 minutes, 2.3 m/s [8.4 km/h]	4.65%
Cook (2017)	Flex - Normal	Wrist	Controlled	Sleep	Polysomnography (Electrical Geodesics)	Min / night	Total Sleep Time	1-night, TST = Time in bed–time awake	9.1%
Cook (2017)	Flex - Normal	Wrist	Controlled	Sleep	Polysomnography (Electrical Geodesics)	%	Sleep Efficiciency (Total Sleep / [illegible]	1-night, SE = TST/time in bedd	8.8%
Cook (2017)	Flex - Normal	Wrist	Controlled	Sleep	Polysomnography (Electrical Geodesics)	Min / night	Sleep Onset Latency	1-night, Bed onset to sleep onset	-11.6%
Cook (2017)	Flex - Normal	Wrist	Controlled	Sleep	Polysomnography (Electrical Geodesics)	Min / night	Wake after Sleep Onset	1-night, awake after sleep onset	-181.1%
Cook (2017)	Flex - Sensitive	Wrist	Controlled	Sleep	Polysomnography (Electrical Geodesics)	Min / night	Total Sleep Time	1-night, TST = Time in bed–time awake	-18.9%
Cook (2017)	Flex - Sensitive	Wrist	Controlled	Sleep	Polysomnography (Electrical Geodesics)	%	Sleep Efficiciency (Total Sleep / [illegible]	1-night, SE = TST/time in bedd	-19.0%
Cook (2017)	Flex - Sensitive	Wrist	Controlled	Sleep	Polysomnography (Electrical Geodesics)	Min / night	Sleep Onset Latency	1-night, Bed onset to sleep onset	59.9%
Cook (2017)	Flex - Sensitive	Wrist	Controlled	Sleep	Polysomnography (Electrical Geodesics)	Min / night	Wake after Sleep Onset	1-night, awake after sleep onset	109.5%
Dannecker (2013)	Classic	Hip	Controlled	Energy Expenditure	Whole-room (Direct) Calorimetry	Total EE (kcal / 3.5 hours)	ADLs and Physical Activity	Random Assigned Activities - Fitbit measured	-27.29%
Diaz (2016)	One	Hip	Controlled	Steps	Visual Observation / Count	Time: Steps / 6 min	Jog: Treadmill	Jog 6 minutes 5.2 mph) (2.32 m/s)	-1.00%
Diaz (2016)	one	Torso	Controlled	Steps	Visual Observation / Count	Time: Steps / 6 min	Jog: Treadmill	Jog 6 minutes 5.2 mph) (2.32 m/s)	-0.20%
Diaz (2016)	flex	Wrist	Controlled	Steps	Visual Observation / Count	Time: Steps / 6 min	Walk: Treadmill	Walk 6 minutes 3.0 mph (1.34 m/s)	-10.00%
Diaz (2016)	one	Torso	Controlled	Steps	Visual Observation / Count	Time: Steps / 6 min	Walk: Treadmill	Walk 6 minutes 1.9 mph (0.85 m/s).	-3.90%
Diaz (2016)	one	Hip	Controlled	Steps	Visual Observation / Count	Time: Steps / 6 min	Walk: Treadmill	Walk 6 minutes 3.0 mph (1.34 m/s)	-0.70%
Diaz (2016)	one	Torso	Controlled	Steps	Visual Observation / Count	Time: Steps / 6 min	Walk: Treadmill	Walk 6 minutes 3.0 mph (1.34 m/s)	-0.60%
Diaz (2016)	one	hip	Controlled	Steps	Visual Observation / Count	Time: Steps / 6 min	Walk: Treadmill	Walk 6 minutes 4.0 mph) (1.79 m/s)	-0.50%
Diaz (2016)	one	Torso	Controlled	Steps	Visual Observation / Count	Time: Steps / 6 min	Walk: Treadmill	Walk 6 minutes 4.0 mph) (1.79 m/s)	-0.50%
Diaz (2016)	flex	wrist	Controlled	Steps	Visual Observation / Count	Time: Steps / 6 min	Jog: Treadmill	Walk 6 minutes 5.2 mph) (2.32 m/s)	2.10%
Diaz (2016)	one	Hip	Controlled	Steps	Visual Observation / Count	Time: Steps / 6 min	Walk: Treadmill	Walk 6 minutes 1.9 mph (0.85 m/s).	-2.00%
Diaz (2016)	flex	Wrist	Controlled	Steps	Visual Observation / Count	Time: Steps / 6 min	Walk: Treadmill	Walk 6 minutes 1.9 mph (0.85 m/s).	-15.30%
Diaz (2016)	flex	Wrist	Controlled	Steps	Visual Observation / Count	Time: Steps / 6 min	Walk: Treadmill	Walk 6 minutes 4.0 mph) (1.79 m/s)	-11.60%

Diaz (2016)	Flex	wrist	Controlled	Energy Expenditure	Indirect calorimetry (Ultima CPX MedGraphics)	EE (kcal/1-min)	Walk: Treadmill	slow walk (1.9 mph (0.85 m/s).	85.71%
Diaz (2016)	One	Torso	Controlled	Energy Expenditure	Indirect calorimetry (Ultima CPX MedGraphics)	EE (kcal/1-min)	Walk: Treadmill	slow walk (1.9 mph (0.85 m/s).	-10.71%
Diaz (2016)	One	hip	Controlled	Energy Expenditure	Indirect calorimetry (Ultima CPX MedGraphics)	EE (kcal/1-min)	Walk: Treadmill	slow walk (1.9 mph (0.85 m/s).	-7.14%
Diaz (2016)	Flex	wrist	Controlled	Energy Expenditure	Indirect calorimetry (Ultima CPX MedGraphics)	EE (kcal/1-min)	Walk: Treadmill	moderate walk (3.0 mph (1.34 m/s)	68.42%
Diaz (2016)	One	Torso	Controlled	Energy Expenditure	Indirect calorimetry (Ultima CPX MedGraphics)	EE (kcal/1-min)	Walk: Treadmill	moderate walk (3.0 mph (1.34 m/s)	15.79%
Diaz (2016)	One	hip	Controlled	Energy Expenditure	Indirect calorimetry (Ultima CPX MedGraphics)	EE (kcal/1-min)	Walk: Treadmill	moderate walk (3.0 mph (1.34 m/s)	7.89%
Diaz (2016)	Flex	wrist	Controlled	Energy Expenditure	Indirect calorimetry (Ultima CPX MedGraphics)	EE (kcal/1-min)	Walk: Treadmill	brisk walk (4.0 mph) (1.79 m/s)	28.57%
Diaz (2016)	One	Torso	Controlled	Energy Expenditure	Indirect calorimetry (Ultima CPX MedGraphics)	EE (kcal/1-min)	Walk: Treadmill	brisk walk (4.0 mph) (1.79 m/s)	14.29%
Diaz (2016)	One	hip	Controlled	Energy Expenditure	Indirect calorimetry (Ultima CPX MedGraphics)	EE (kcal/1-min)	Walk: Treadmill	brisk walk (4.0 mph) (1.79 m/s)	7.14%
Diaz (2016)	Flex	wrist	Controlled	Energy Expenditure	Indirect calorimetry (Ultima CPX MedGraphics)	EE (kcal/1-min)	Jog: Treadmill	Jog (5.2 mph) (2.32 m/s)	27.71%
Diaz (2016)	One	Torso	Controlled	Energy Expenditure	Indirect calorimetry (Ultima CPX MedGraphics)	EE (kcal/1-min)	Jog: Treadmill	Jog (5.2 mph) (2.32 m/s)	14.46%
Diaz (2016)	One	hip	Controlled	Energy Expenditure	Indirect calorimetry (Ultima CPX MedGraphics)	EE (kcal/1-min)	Jog: Treadmill	Jog (5.2 mph) (2.32 m/s)	-4.82%
Dominick (2016)	Flex	Wrist	Freeliving	Steps	Accelerometry (Actigraph GT3X)	Steps / day	Normal Daility Activities	14 days (2 7-day sessions, waking hours)	6.7%
Dondzila (2016)	Charge	Wrist	Controlled	Energy Expenditure	Indirect calorimetry (TrueOne 2400 metabolic	Total EE (kcals / 5 min)	Walk: Treadmill	walking, 80.5 m/min [1.34 m/s]	21.40%
Dondzila (2016)	Charge	Wrist	Controlled	Energy Expenditure	Indirect calorimetry (TrueOne 2400 metabolic	Total EE (kcals / 5 min)	Walk: Treadmill	walking, 107.3 m/min [1.79 m/s]	-11.20%
Dondzila (2016)	Charge	Wrist	Controlled	Energy Expenditure	Indirect calorimetry (TrueOne 2400 metabolic	Total EE (kcals / 5 min)	Jog: Treadmill	Jog 134.1 m/min [2.24 m/s]	-13.73%
Dondzila (2016)	Charge	Wrist	Controlled	Energy Expenditure	Indirect calorimetry (TrueOne 2400 metabolic	Total EE (kcals / 5 min)	Run: Treadmill	Run 160.9 m/min (6.0 mph) [2.68 m/s]	-22.45%
Dooley (2017)	Charge HR	Wrist	Controlled	Energy Expenditure	Indirect calorimetry (ParvoMedics TrueOne 2400)	kcal/10 min	Baseline/Rest	10 minutes, Rest	-9.02%
Dooley (2017)	Charge HR	Wrist	Controlled	Energy Expenditure	Indirect calorimetry (ParvoMedics TrueOne 2400)	kcal/4 min	Walk Treadmill: 2.5mph 1.12	4 minutes, 1.11 m/s	82.27%
Dooley (2017)	Charge HR	Wrist	Controlled	Energy Expenditure	Indirect calorimetry (ParvoMedics TrueOne 2400)	kcal/4 min	Walk Treadmill: 3.5 mph 1.56	4 minutes, 1.56 m/s	42.20%
Dooley (2017)	Charge HR	Wrist	Controlled	Energy Expenditure	Indirect calorimetry (ParvoMedics TrueOne 2400)	kcal/4 min	Walk Treadmill: 5.5 mph 2.5	4 minutes, 2.46 m/s	2.84%
Dooley (2017)	Charge HR	Wrist	Controlled	Energy Expenditure	Indirect calorimetry (ParvoMedics TrueOne 2400)	kcal/10 min	Recovery/Rest	10 minutes, Rest	18.96%
Farina (2017)	Charge HR	Wrist	Freeliving	Steps	Accelerometry (Actigraph GT3X))	Steps / day	Normal Daility Activities	8 days, waking hours	-36%
Ferguson (2012)	Zip	Hip	Freeliving	Time in Activity	Accelerometry (Actigraph GT3X))	MVPA (Mins / Day)	2-Day, 48 hour (24 hour wear)	AG not defined ZIP sum of vigorous and moderate minutes.	146%
Ferguson (2012)	One	Hip	Freeliving	Time in Activity	Accelerometry (Actigraph GT3X))	MVPA (Mins / Day)	2-Day, 48 hour (24 hour wear)	AG not defined ONE sum of very active and moderate minutes.	113%
Ferguson (2012)	Zip	Hip	Free Living	Energy Expenditure	Accelerometry (SenseWear model MF)	Total EE (kcal / day)	Normal Daily Activities	2-Days	-16.5%
Ferguson (2012)	One	Hip	Free Living	Energy Expenditure	Accelerometry (SenseWear model MF)	Total EE (kcal / day)	Normal Daily Activities	2-Days	-15.8%
Ferguson (2015)	Zip	Hip	Freeliving	Steps	Accelerometry (Actigraph GT3X))	Steps / day	Normal Daility Activities	2-Day, 48 hour (24 hour wear)	4.4%
Ferguson (2015)	One	Hip	Freeliving	Steps	Accelerometry (Actigraph GT3X))	Steps / day	Normal Daility Activities	2-Day, 48 hour (24 hour wear)	5.6%
Ferguson (2015)	One	Wrist	Free Living	Sleep	Accelerometry (SW Mini)	Total Bed Time (min)	Home sleep	2-nights	3.80%
Floegel (2016)	One	Hip	Controlled	Steps	Visual Observation / Count	Distance: Steps / 100 m	Walk: Hallway	100m self-selected pace (walker user)	-23.25%
Floegel (2016)	One	Hip	Controlled	Steps	Visual Observation / Count	Distance: Steps / 100 m	Walk: Hallway	100m self-selected pace (impaired)	-1.71%
Floegel (2016)	One	Hip	Controlled	Steps	Visual Observation / Count	Distance: Steps / 100 m	Walk: Hallway	100m self-selected pace (cane-users)	-11.48%
Floegel (2016)	Flex	Wrist	Controlled	Steps	Visual Observation / Count	Distance: Steps / 100 m	Walk: Hallway	100m self-selected pace (walker user)	-57.40%
Floegel (2016)	Flex	Wrist	Controlled	Steps	Visual Observation / Count	Distance: Steps / 100 m	Walk: Hallway	100m self-selected pace (cane-users)	-22.88%
Floegel (2016)	Flex	Wrist	Controlled	Steps	Visual Observation / Count	Distance: Steps / 100 m	Walk: Hallway	100m self-selected pace (impaired)	-16.31%
Floegel (2016)	One	Hip	Controlled	Steps	Visual Observation / Count	Distance: Steps / 100 m	Walk: Hallway	100m self-selected pace (non-impaired)	-2.59%
Floegel (2016)	Flex	Wrist	Controlled	Steps	Visual Observation / Count	Distance: Steps / 100 m	Walk: Hallway	100m self-selected pace (non-impaired)	-26.94%
Fokkema (2016)	Charge HR	Wrist	Controlled	Steps	Visual Observation / Count	Time: Steps/ 30 min	Jog: Treadmill	fast: 6.4 km/h, 1.78 m/s	5.18%
Fokkema (2016)	Charge HR	Wrist	Controlled	Steps	Visual Observation / Count	Time: Steps/ 30 min	Walk: Treadmill	comfortable: 4.8 km/h, 1.33 m/s	1.98%
Fokkema (2016)	Charge HR	Wrist	Controlled	Steps	Visual Observation / Count	Time: Steps/ 30 min	Walk: Treadmill	slow: 3.2 km/h, 0.89 m/s	-0.74%
Fulk (2014)	Ultra	Hip	Controlled	Steps	Visual Observation / Count	Time: steps / 2 min	Walk: Hallway	2 Minute walk test (steps / 2 minutes) - Stroke (n=30)	-8.35%
Fulk (2014)	Ultra	Hip	Controlled	Steps	Visual Observation / Count	Time: steps / 2 min	Walk: Hallway	2 Minute walk test (steps / 2 minutes) - Able (n=20)	-0.15%
Gomersall (2016)	One	Hip	Freeliving	Steps	Accelerometry (Actigraph GT3X)	Steps / day	Normal Daility Activities	9.6 days (waking hours)	7.9%
Gomersall (2016)	One	hip	Freeliving	Time in Activity	Accelerometry (Actigraph GT3X)	MVPA (Min / Day)	9.6 days (waking hours)	MVPA: > = 2020 counts / minute. FB Very Active minutes	76%
Gusmer (2014)	Ultra	Hip	Controlled	Energy Expenditure	Indirect calorimetry (Ultima CPX metabolic cart)	Total EE (kcal / 30-min)	Walk: Treadmill	Slow Walk (-10% of their self-selected comfortable walking speed)	-12.78%
Gusmer (2014)	Ultra	Hip	Controlled	Energy Expenditure	Indirect calorimetry (Ultima CPX metabolic cart)	Total EE (kcal / 30-min)	Walk: Treadmill	Fast Walk (+10% of their self-selected comfortable walking speed)	-17.23%
Hargens (2017)	Charge	Wrist	Freeliving	Steps	Accelerometry (Actigraph GT3X))	Steps / day	Normal Daility Activities	7 days, waking hours	17.9%
Hargens (2017)	One	Waist	Freeliving	Steps	Accelerometry (Actigraph GT3X))	Steps / day	Normal Daility Activities	7 days, waking hours	10.2%
Huang, (2016)	One	Waist	Controlled	Steps	Visual Observation / Count	Stair: Steps / 176 stairs	Stair: Down	Downstairs (176 steps) self selected speed	-6.00%

Huang, (2016)	Zip	Waist	Controlled	Steps	Visual Observation / Count	Stair: Steps / 176 stairs	Stair: Down	Downstairs (176 steps) self selected speed	-5.30%
Huang, (2016)	One	waist	Controlled	Steps	Visual Observation / Count	Stair: Steps / 176 stairs	Stair: Up	Upstairs (176 steps) self selected speed	-2.30%
Huang, (2016)	Zip	Waist	Controlled	Steps	Visual Observation / Count	Stair: Steps / 176 stairs	Stair: Up	Upstairs (176 steps) self selected speed	-1.10%
Huang, (2016)	Flex	Wrist	Controlled	Steps	Visual Observation / Count	Stair: Steps / 176 stairs	Stair: Up	Upstairs (176 steps) self selected speed	-7.90%
Huang, (2016)	Flex	Wrist	Controlled	Steps	Visual Observation / Count	Stair: Steps / 176 stairs	Stair: Down	Downstairs (176 steps) self selected speed	-7.10%
Huang, (2016)	Zip	Waist	Controlled	Steps	Visual Observation / Count	Time: Steps / 3 min	Walk: Treadmill	(1.33 m/s), 3 min	-5.80%
Huang, (2016)	Zip	Waist	Controlled	Steps	Visual Observation / Count	Time: Steps / 3 min	Walk: Treadmill	(1.78 m/s), 3 min	-3.40%
Huang, (2016)	One	Waist	Controlled	Steps	Visual Observation / Count	Time: Steps / 3 min	Walk: Treadmill	(1.78 m/s), 3 min	-1.50%
Huang, (2016)	One	Waist	Controlled	Steps	Visual Observation / Count	Time: Steps / 3 min	Walk: Treadmill	(1.33 m/s), 3 min	-1.20%
Huang, (2016)	One	Waist	Controlled	Steps	Visual Observation / Count	Time: Steps / 3 min	Walk: Treadmill	(0.9 m/s), 3 min	-3.80%
Huang, (2016)	Zip	Waist	Controlled	Steps	Visual Observation / Count	Time: Steps / 3 min	Walk: Treadmill	(0.9 m/s), 3 min	-2.70%
Huang, (2016)	One	Waist	Controlled	Steps	Visual Observation / Count	Distance: Steps / 400 m	Walk: outside, paved road	400m), self selected speed	-1.00%
Huang, (2016)	Zip	Waist	Controlled	Steps	Visual Observation / Count	Distance: Steps / 400 m	Walk: outside, paved road	400m, self selected speed	-0.10%
Huang, (2016)	Flex	Wrist	Controlled	Steps	Visual Observation / Count	Distance: Steps / 400 m	Walk: outside, paved road	400m, self selected speed	-11.70%
Huang, (2016)	Flex	Wrist	Controlled	Steps	Visual Observation / Count	Time: Steps / 3 min	Walk: Treadmill	(1.78 m/s), 3 min	-8.90%
Huang, (2016)	Flex	Wrist	Controlled	Steps	Visual Observation / Count	Time: Steps / 3 min	Walk: Treadmill	(1.33 m/s), 3 min	-5.10%
Huang, (2016)	Flex	Wrist	Controlled	Steps	Visual Observation / Count	Time: Steps / 3 min	Walk: Treadmill	(0.9 m/s), 3 min	-6.54%
Huang, (2016)	One	Waist	Controlled	Distance	Treadmill Distance	Distance / 3 min	Walk: Treadmill	(0.9 m/s), 3 min	31.20%
Huang, (2016)	Zip	Waist	Controlled	Distance	Treadmill Distance	Distance / 3 min	Walk: Treadmill	(0.9 m/s), 3 min	29.90%
Huang, (2016)	Flex	Wrist	Controlled	Distance	Treadmill Distance	Distance / 3 min	Walk: Treadmill	(0.9 m/s), 3 min	26.80%
Huang, (2016)	One	Waist	Controlled	Distance	Treadmill Distance	Distance / 3 min	Walk: Treadmill	(1.33 m/s), 3 min	3.70%
Huang, (2016)	Zip	Waist	Controlled	Distance	Treadmill Distance	Distance / 3 min	Walk: Treadmill	(1.33 m/s), 3 min	-1.80%
Huang, (2016)	Flex	Wrist	Controlled	Distance	Treadmill Distance	Distance / 3 min	Walk: Treadmill	(1.33 m/s), 3 min	-2.60%
Huang, (2016)	One	Waist	Controlled	Distance	Treadmill Distance	Distance / 3 min	Walk: Treadmill	(1.78 m/s), 3 min	-12.10%
Huang, (2016)	Zip	Waist	Controlled	Distance	Treadmill Distance	Distance / 3 min	Walk: Treadmill	(1.78 m/s), 3 min	-7.70%
Huang, (2016)	Flex	Wrist	Controlled	Distance	Treadmill Distance	Distance / 3 min	Walk: Treadmill	(1.78 m/s), 3 min	-19.60%
Huang, (2016)	One	Waist	Controlled	Distance	Measured Distance	Distance: 400 m	Walk: outside, paved road	400 m, self selected speed	5.40%
Huang, (2016)	Zip	Waist	Controlled	Distance	Measured Distance	Distance: 400 m	Walk: outside, paved road	400 m, self selected speed	4.10%
Huang, (2016)	Flex	Waist	Controlled	Distance	Measured Distance	Distance: 400 m	Walk: outside, paved road	400 m, self selected speed	12.80%
Hui (2017)	One	Ankle (non	Freeliving	Steps	Accelerometry (Actical)	Steps / day	Normal Daility Activities	3 days (1 weekday, 2 weekend), 24 hours	-5%
Hui (2017)	One	Ankle (non	Freeliving	Time in Activity	Accelerometry (Actical)	Sedentary (min / day)	3 days (1 weekday, 2	AC: sed 1 MET, lignt <3 MET, Mod 3-6 MET, Vig >6 METS	-4%
Hui (2017)	One	Ankle (non	Freeliving	Time in Activity	Accelerometry (Actical)	Light (Mins / Day)	3 days (1 weekday, 2	AC: sed 1 MET, lignt <3 MET, Mod 3-6 MET, Vig >6 METS	47%
Hui (2017)	One	Ankle (non	Freeliving	Time in Activity	Accelerometry (Actical)	Moderate (Min / Day),	3 days (1 weekday, 2	AC: sed 1 MET, lignt <3 MET, Mod 3-6 MET, Vig >6 METS	-74%
Hui (2017)	One	Ankle (non	Freeliving	Time in Activity	Accelerometry (Actical)	Vigorous (Min / Day),	3 days (1 weekday, 2	AC: sed 1 MET, lignt <3 MET, Mod 3-6 MET, Vig >6 METS	1900%
Imboden (2017)	Zip	waist	Controlled	Steps	Visual Observation / Count	Time: Steps / 80 min	Mixed Progressive Intensity	total steps/80 min protocol (semi structured, >12 out of 21 choices for:	-26.28%
Imboden (2017)	One	waist	Controlled	Steps	Visual Observation / Count	Time: Steps / 80 min	Mixed Progressive Intensity	total steps/80 min protocol (semi structured, >12 out of 21 choices for:	-26.09%
Imboden (2017)	Flex	Wrist	Controlled	Steps	Visual Observation / Count	Time: Steps / 80 min	Mixed Progressive Intensity	total steps/80 min protocol (semi structured, >12 out of 21 choices for:	-23.06%
Imboden (2017)	Flex	Wrist	Controlled	Energy Expenditure	Indirect calorimetry (COSMED Srl):	kcal/80 min	ADLs protocol - Progressive	Progressive Intensity ADL simulation (sedentary activities, household	-13.27%
Imboden (2017)	One	Waist	Controlled	Energy Expenditure	Indirect calorimetry (COSMED Srl):	kcal/80 min	ADLs protocol - Progressive	Progressive Intensity ADL simulation (sedentary activities, household	-15.99%
Imboden (2017)	Zip	Waist	Controlled	Energy Expenditure	Indirect calorimetry (COSMED Srl):	kcal/80 min	ADLs protocol - Progressive	Progressive Intensity ADL simulation (sedentary activities, household	-26.19%
Klassen (2015)	One	Ankle	Controlled	Steps	Visual Observation / Count	Distance: Steps / 15 m	Walk: Hallway	15 m walk, 0.9m/s	-3.45%
Klassen (2015)	One	Ankle	Controlled	Steps	Visual Observation / Count	Distance: Steps / 15 m	Walk: Hallway	15 m walk, 0.8m/s	0.00%
Klassen (2015)	One	Waist	Controlled	Steps	Visual Observation / Count	Distance: Steps / 15 m	Walk: Hallway	15 m walk, 0.8m/s	-9.68%
Klassen (2015)	One	Waist	Controlled	Steps	Visual Observation / Count	Distance: Steps / 15 m	Walk: Hallway	15 m walk, 0.9m/s	-6.90%
Klassen (2015)	One	Ankle	Controlled	Steps	Visual Observation / Count	Distance: Steps / 15 m	Walk: Hallway	15 m walk, 0.3m/s	-9.26%
Klassen (2015)	One	Ankle	Controlled	Steps	Visual Observation / Count	Distance: Steps / 15 m	Walk: Hallway	15 m walk, 0.7m/s	-3.03%
Klassen (2015)	One	Ankle	Controlled	Steps	Visual Observation / Count	Distance: Steps / 15 m	Walk: Hallway	15 m walk, 0.4m/s	-2.17%
Klassen (2015)	One	Ankle	Controlled	Steps	Visual Observation / Count	Distance: Steps / 15 m	Walk: Hallway	15 m walk, 0.5m/s	0.00%
Klassen (2015)	One	Ankle	Controlled	Steps	Visual Observation / Count	Distance: Steps / 15 m	Walk: Hallway	15 m walk, 0.6m/s	0.00%
Klassen (2015)	One	Waist	Controlled	Steps	Visual Observation / Count	Distance: Steps / 15 m	Walk: Hallway	15 m walk, 0.3m/s	-81.48%

Klassen (2015)	One	Waist	Controlled	Steps	Visual Observation / Count	Distance: Steps / 15 m	Walk: Hallway	15 m walk, 0.4m/s	-56.52%
Klassen (2015)	One	Waist	Controlled	Steps	Visual Observation / Count	Distance: Steps / 15 m	Walk: Hallway	15 m walk, 0.5m/s	-35.00%
Klassen (2015)	One	Waist	Controlled	Steps	Visual Observation / Count	Distance: Steps / 15 m	Walk: Hallway	15 m walk, 0.6m/s	-13.89%
Klassen (2015)	One	Waist	Controlled	Steps	Visual Observation / Count	Distance: Steps / 15 m	Walk: Hallway	15 m walk, 0.7m/s	-12.12%
Klassen (2015)	Zip	Hip	Controlled	Steps	Visual Observation / Count	Time: Step / 30 min	Walk: Treadmil	30 minute (x2)s, 4.3 km / hr [1.2 m/s]	-0.33%
Klassen (2015)	Flex	Wrist	Controlled	Steps	Visual Observation / Count	Time: Step / 30 min	Walk: Treadmil	30 minute (x2)s, 4.3 km / hr [1.2 m/s]	-5.67%
Kooiman (2015)	Flex	Wrist	Freeliving	Steps	Accelerometry (ActivePAL)	Steps / 7.5 hr	Normal Dailiy Activities	9 am to 4:40 pm, 1-day	7.4%
Kooiman (2015)	Zip	Hip	Freeliving	Steps	Accelerometry (ActivePAL)	Steps / 7.5 hr	Normal Dailiy Activities	9 am to 4:40 pm, 1-day	1.2%
Lee (2014)	One	Hip	Controlled	Energy Expenditure	Indirect calorimetry (Oxycon Mobile)	Total EE (kcal / 69-min)	Physical Activity: Progressive	Sedentary x4, walking x4, running x2, MVPA x6	-7.3%
Lee (2014)	Zip	Hip	Controlled	Energy Expenditure	Indirect calorimetry (Oxycon Mobile)	Total EE (kcal / 69-min)	Physical Activity: Progressive	Sedentary x4, walking x4, running x2, MVPA x5	3.70%
Lee (2017)	Charge	Wrist	Free Living	Sleep	Accelerometry (Actiwatch)	Total Bed Time (min)	Home sleep	13-days	-0.4%
Lee (2017)	Charge	Wrist	Free Living	Sleep	Accelerometry (Actiwatch)	Total Sleep Time (min)	Home sleep	13-days	5.8%
Mantura (2016)	Flex - Normal	Wrist	Controlled	Sleep	Polysomnography (Aura system)	Min / night	Total Sleep	1-night, Total Minutes defined as sleep	3.0%
Mantura (2016)	Flex - Normal	Wrist	Controlled	Sleep	Polysomnography (Aura system)	%	Sleep Efficiciency (Total Sleep /	1-night, Total minutes of sleep / total minutes in bed	11.6%
Middelweerd (2017)	One	Hip	Freeliving	Steps	Accelerometry (Actigraph GT3X))	Steps / day	Normal Dailiy Activities	7 days, waking hours	8.9%
Middelweerd (2017)	One	Hip	Freeliving	Time in Activity	Accelerometry (Actigraph GT3X))	Moderate (Min / Day),	7 days, waking hours	AG: < 100, 100-2019, 2020-5998, >=5999; MVPA >=2020	124%
Middelweerd (2017)	One	Hip	Freeliving	Time in Activity	Accelerometry (Actigraph GT3X))	Vigorous (Min / Day)	7 days, waking hours	AG: < 100, 100-2019, 2020-5998, >=5999; MVPA >=2020	358%
Middelweerd (2017)	One	Hip	Freeliving	Time in Activity	Accelerometry (Actigraph GT3X))	MVPA (Mins / Day)	7 days, waking hours	AG: < 100, 100-2019, 2020-5998, >=5999; MVPA >=2020	150%
Modave (2017)	Surge	Wrist	Controlled	Steps	Visual Observation / Count	Number: #/1000	Walking: Hallway	1000 steps, 0.89 m/s to 0.34 m/s (self-selected), age 18-39	-4.03%
Modave (2017)	Surge	Wrist	Controlled	Steps	Visual Observation / Count	Number: #/1000	Walking: Hallway	1000 steps, 0.89 m/s to 0.34 m/s (self-selected), age 40-64	-5.61%
Montes (2017)	Flex	Wrist	Controlled	Steps	Visual Observation / Count	Time: Steps/3 min	Walking: Treadmill	steps/3 min, 1.5 mph, 0.67 m/s	-14.13%
Montes (2017)	Flex	Wrist	Controlled	Steps	Visual Observation / Count	Time: Steps/3 min	Walking: Treadmill	steps/3 min, 2.5 mph, 1.12 m/s	-2.71%
Montes (2017)	Flex	Wrist	Controlled	Steps	Visual Observation / Count	Time: Steps/3 min	Walking: Treadmill	steps/3 min, 3.5 mph, 1.56 m/s	-3.68%
Montes (2017)	Flex	Wrist	Controlled	Energy Expenditure	Indirect calorimetry (MOXUS Srl):	kcal/3 min	Walk: Treadmill	0.67 m/s (Slow Walk) [1.5 mph]	63.28%
Montes (2017)	Flex	Wrist	Controlled	Energy Expenditure	Indirect calorimetry (MOSUX Srl):	kcal/3 min	Walk: Treadmill	1.11 m/s (normal walk) [2.5 mph]	73.25%
Montes (2017)	Flex	Wrist	Controlled	Energy Expenditure	Indirect calorimetry (MOXUS Srl):	kcal/3 min	Walk: Treadmill	1.56 m/s (normal walk) [3.5 mph]	39.99%
Montgomery (2012)	Likely One	Wrist	Controlled	Sleep	Polysomnography (Embla Systems)	Min / night	Total Sleep Time	1-night, TST = Time in bed–time awake	18.1%
Montgomery (2012)	Likely One	Wrist	Controlled	Sleep	Polysomnography (Embla Systems)	%	Sleep Efficiciency (Total Sleep /	1-night, SE = TST/time in bedd	18.2%
Montoye (2017)	Charge HR	Wrist	Controlled	Energy Expenditure	Indirect Calorimetry (TrueOne 2400)	kcal/1 min (x5)	Lie down	Lie down	-5.26%
Montoye (2017)	Charge HR	Wrist	Controlled	Energy Expenditure	Indirect Calorimetry (TrueOne 2400)	kcal/1 min (x5)	Sit	Sit	1.41%
Montoye (2017)	Charge HR	Wrist	Controlled	Energy Expenditure	Indirect Calorimetry (TrueOne 2400)	kcal/1 min (x5)	Stand	Stand	0.00%
Montoye (2017)	Charge HR	Wrist	Controlled	Energy Expenditure	Indirect Calorimetry (TrueOne 2400)	kcal/1 min (x5)	Walk: Treadmill	0.89 m/s	4.68%
Montoye (2017)	Charge HR	Wrist	Controlled	Energy Expenditure	Indirect Calorimetry (TrueOne 2400)	kcal/1 min (x5)	Walk Incline: Treadmill	0.89 m/s 5% grade	9.29%
Montoye (2017)	Charge HR	Wrist	Controlled	Energy Expenditure	Indirect Calorimetry (TrueOne 2400)	kcal/1 min (x5)	Walk Incline: Treadmill	0.89 m/s 10% grade	10.03%
Montoye (2017)	Charge HR	Wrist	Controlled	Energy Expenditure	Indirect Calorimetry (TrueOne 2400)	kcal/1 min (x5)	Walk: Treadmill	1.34 m/s 0% grade	4.93%
Montoye (2017)	Charge HR	Wrist	Controlled	Energy Expenditure	Indirect Calorimetry (TrueOne 2400)	kcal/1 min (x5)	Walk Incline: Treadmill	1.34 m/s 5% grade	9.24%
Montoye (2017)	Charge HR	Wrist	Controlled	Energy Expenditure	Indirect Calorimetry (TrueOne 2400)	kcal/1 min (x5)	Walk: Treadmill	1.56 m/s to 1.8 m/s	7.60%
Montoye (2017)	Charge HR	Wrist	Controlled	Energy Expenditure	Indirect Calorimetry (TrueOne 2400)	kcal/1 min (x5)	Jog	2.24/4.5 m/s	12.83%
Montoye (2017)	Charge HR	Wrist	Controlled	Energy Expenditure	Indirect Calorimetry (TrueOne 2400)	kcal/1 min (x5)	Cycle	75/150 W	8.41%
Murakami (2016)	Flex	Wrist	Metabolic	Energy Expenditure	Whole-room (Direct) Calorimetry	Total EE (kcals / day)	Normal Daily Activities:	3 meals, deskwork, watching TV, housework, treadmill	6.03%
Murakami (2016)	Flex	Wrist	Free Living	Energy Expenditure	Doubly Labeled Water	Total EE (kcals / day)	Normal Daily Activities	15 -Days	-7.4%
Nelson (2016)	One	Hip	Controlled	Steps	Visual Observation / Count	Time: Steps / 20 min	ADL	Household activities (4 x 5 min)	-63.50%
Nelson (2016)	Zip	Hip	Controlled	Steps	Visual Observation / Count	Time: Steps / 20 min	ADL	Household activities (4 x 5 min)	-62.60%
Nelson (2016)	Flex	Wrist	Controlled	Steps	Visual Observation / Count	Time: Steps / 20 min	ADL	Household activities (4 x 5 min)	-8.10%
Nelson (2016)	One	Hip	Controlled	Steps	Visual Observation / Count	Time: Steps / 20 min	Ambuation: Hallway or	Ambulatory activiities (4 x 5 min)	1.40%
Nelson (2016)	Zip	Hip	Controlled	Steps	Visual Observation / Count	Time: Steps / 20 min	Ambuation: Hallway or	Ambulatory activiities (4 x 5 min)	2.00%
Nelson (2016)	Flex	Wrist	Controlled	Steps	Visual Observation / Count	Time: Steps / 20 min	Ambuation: Hallway or	Ambulatory activiities (4 x 5 min)	-3.60%
Nelson (2016)	Zip	hip	Controlled	Energy Expenditure	Indirect calorimetry (COSMED Srl):	Total EE (kcals / 20min)	ADLs	Household activities (standing, dusting, sweeping, vacuuming, folding	-34.17%
Nelson (2016)	One	hip	Controlled	Energy Expenditure	Indirect calorimetry (COSMED Srl):	Total EE (kcals / 20min)	ADLs	Household activities (standing, dusting, sweeping, vacuuming, folding	-26.64%
Nelson (2016)	Flex	wrist	Controlled	Energy Expenditure	Indirect calorimetry (COSMED Srl):	Total EE (kcals / 20min)	ADLs	Household activities (standing, dusting, sweeping, vacuuming, folding	-7.25%

Nelson (2016)	One	hip	Controlled	Energy Expenditure	Indirect calorimetry (COSMED Srl):	Total EE (kcals / 20 min)	Ambuation: Hallway or	Ambulatory activiities x 4 slow overground walk, brisk overground walk,	15.95%
Nelson (2016)	Zip	hip	Controlled	Energy Expenditure	Indirect calorimetry (COSMED Srl):	Total EE (kcals / 20 min)	Ambuation: Hallway or	Ambulatory activiities x 4 slow overground walk, brisk overground walk,	39.51%
Nelson (2016)	Flex	wrist	Controlled	Energy Expenditure	Indirect calorimetry (COSMED Srl):	Total EE (kcals / 20 min)	Ambuation: Hallway or	Ambulatory activiities x 4 slow overground walk, brisk overground walk,	32.63%
Nelson (2016)	One	hip	Controlled	Energy Expenditure	Indirect calorimetry (COSMED Srl):	Total EE (kcals / 15 min)	Resting	Sedentary activities x 3 (lying down, watching television, writing, reading,	-6.82%
Nelson (2016)	Zip	hip	Controlled	Energy Expenditure	Indirect calorimetry (COSMED Srl):	Total EE (kcals / 15 min)	Resting	Sedentary activities x 3 (lying down, watching television, writing, reading,	-2.92%
Nelson (2016)	Flex	wrist	Controlled	Energy Expenditure	Indirect calorimetry (COSMED Srl):	Total EE (kcals / 15 min)	Resting	Sedentary activities x 3 (lying down, watching television, writing, reading,	-6.82%
O'Connell (2016)	One	Chest	Controlled	Steps	Visual Observation / Count	Stair: Steps / 49 stairs	Stairs: Up	Stairs up (49 steps, self selected pace) step height 16cm	-0.90%
O'Connell (2016)	One	Chest	Controlled	Steps	Visual Observation / Count	Stair: Steps / 49 stairs	Stairs: Down	Stairs down (49 steps, self-selected pace) step height 16cm	0.90%
O'Connell (2016)	One	Chest	Controlled	Steps	Visual Observation / Count	Distance: Steps / 880 m	Walk: Ashphalt	Tarmacadam/asphalt (880m) self selected pace	-1.10%
O'Connell (2016)	One	Chest	Controlled	Steps	Visual Observation / Count	Distance: Steps / 900 m	Walk: Grass	Natural lawn grass (900m) self selected pace	-0.50%
O'Connell (2016)	One	Chest	Controlled	Steps	Visual Observation / Count	Distance: Steps / 990 m	Walk: Gravel	gravel (990 m) self selected pace	0.08%
O'Connell (2016)	One	Chest	Controlled	Steps	Visual Observation / Count	Distance: Steps / 800 m	Walk: Linoleum	Linoleum (800 m) self-slected pace	0.30%
O'Connell (2016)	One	Chest	Controlled	Steps	Visual Observation / Count	Distance: Steps / 500 m	Walk: Ramp	Ramp (240 m up, 240 m down, incline of 4.05%) self-selected pace	0.50%
O'Connell (2016)	One	Chest	Controlled	Steps	Visual Observation / Count	Distance: Steps / 400 m	Walk: Ceramic	Ceramic tile (400m) self selected pace	0.90%
Park (2014)	Ultra	Hip	Controlled	Steps	Visual Observation / Count	Time: Steps / min	Jog: Treadmil	6 minutes, @ 161 m/min [2.7 m/s]	-42.24%
Park (2014)	Ultra	Hip	Controlled	Steps	Visual Observation / Count	Time: Steps / min	Jog: Treadmil	6 minutes, @ 134 m/min [2.2 m/s]	-30.81%
Park (2014)	Ultra	Waist	Controlled	Steps	Visual Observation / Count	Time: Steps / min	Jog: Treadmil	6 minutes, @ 161 m/min [2.7 m/s]	-9.33%
Park (2014)	Ultra	Upper Arm /	Controlled	Steps	Visual Observation / Count	Time: Steps / min	Jog: Treadmil	6 minutes, @ 161 m/min [2.7 m/s]	-4.90%
Park (2014)	Ultra	Waist	Controlled	Steps	Visual Observation / Count	Time: Steps / min	Jog: Treadmil	6 minutes, @ 134 m/min [2.2 m/s]	-4.89%
Park (2014)	Ultra	Chest / Lanyard	Controlled	Steps	Visual Observation / Count	Time: Steps / min	Jog: Treadmil	6 minutes, @ 161 m/min [2.7 m/s]	-3.85%
Park (2014)	Ultra	Upper Arm /	Controlled	Steps	Visual Observation / Count	Time: Steps / min	Jog: Treadmil	6 minutes, @ 134 m/min [2.2 m/s]	-3.73%
Park (2014)	Ultra	Chest / Lanyard	Controlled	Steps	Visual Observation / Count	Time: Steps / min	Jog: Treadmil	6 minutes, @ 134 m/min [2.2 m/s]	-2.44%
Park (2014)	Ultra	Hip Pocket	Controlled	Steps	Visual Observation / Count	Time: Steps / min	Walk: Treadmil	6 minutes, @ 107 m/min [1.8 m/s]	-5.75%
Park (2014)	Ultra	Chest / Lanyard	Controlled	Steps	Visual Observation / Count	Time: Steps / min	Walk: Treadmil	6 minutes, @ 107 m/min [1.8 m/s]	-2.91%
Park (2014)	Ultra	Hip	Controlled	Steps	Visual Observation / Count	Time: Steps / min	Walk: Treadmil	6 minutes, @ 54 m/min [.9 m/s]	-1.57%
Park (2014)	Ultra	Upper Arm /	Controlled	Steps	Visual Observation / Count	Time: Steps / min	Walk: Treadmil	6 minutes, @ 107 m/min [1.8 m/s]	-0.82%
Park (2014)	Ultra	Waist	Controlled	Steps	Visual Observation / Count	Time: Steps / min	Walk: Treadmil	6 minutes, @ 80 m/min [1.3 m/s]	-0.69%
Park (2014)	Ultra	Hip	Controlled	Steps	Visual Observation / Count	Time: Steps / min	Walk: Treadmil	6 minutes, @ 80 m/min [1.3 m/s]	-0.52%
Park (2014)	Ultra	Waist	Controlled	Steps	Visual Observation / Count	Time: Steps / min	Walk: Treadmil	6 minutes, @ 107 m/min [1.8 m/s]	-0.15%
Park (2014)	Ultra	Upper Arm /	Controlled	Steps	Visual Observation / Count	Time: Steps / min	Walk: Treadmil	6 minutes, @ 80 m/min [1.3 m/s]	0.00%
Park (2014)	Ultra	Chest / Lanyard	Controlled	Steps	Visual Observation / Count	Time: Steps / min	Walk: Treadmil	6 minutes, @ 54 m/min [.9 m/s]	-0.79%
Park (2014)	Ultra	Chest / Lanyard	Controlled	Steps	Visual Observation / Count	Time: Steps / min	Walk: Treadmil	6 minutes, @ 80 m/min [1.3 m/s]	0.26%
Park (2014)	Ultra	Waist	Controlled	Steps	Visual Observation / Count	Time: Steps / min	Walk: Treadmil	6 minutes, @ 54 m/min [.0.9 m/s]	-0.29%
Park (2014)	Ultra	Upper Arm /	Controlled	Steps	Visual Observation / Count	Time: Steps / min	Walk: Treadmil	6 minutes, @ 54 m/min [.9 m/s]	-0.20%
Park (2014)	Ultra	Upper Arm /	Controlled	Steps	Visual Observation / Count	Time: Steps / min	Walk: Outside	9 minutes, @ self-selected speed	-2.86%
Park (2014)	Ultra	Hip	Controlled	Steps	Visual Observation / Count	Time: Steps / min	Walk: Outside	9 minutes, @ self-selected speed	-2.45%
Park (2014)	Ultra	Chest / Lanyard	Controlled	Steps	Visual Observation / Count	Time: Steps / min	Walk: Outside	9 minutes, @ self-selected speed	-2.18%
Park (2014)	Ultra	Waist	Controlled	Steps	Visual Observation / Count	Time: Steps / min	Walk: Outside	9 minutes, @ self-selected speed	-1.90%
Park (2014)	One, Zip	Hip	Controlled	Steps	Visual Observation / Count	Time: Steps / min	Walk: Hallway	2-minute WT (steps / 2-minutes)	-0.53%
Philips (2015)	Classic	Hip	Controlled	Steps	Visual Observation / Count	Distance: Steps / 100 ft	Walk: Hallway	100 Feet, normal pace	-39.24%
Price (2016)	One	Waist	Controlled	Energy Expenditure	Indirect calorimetry (TrueOne 2400 metabolic	EE (kcal/min)	Treadmill	0.7 m/s	33.84%
Price (2016)	One	Waist	Controlled	Energy Expenditure	Indirect calorimetry (TrueOne 2400 metabolic	EE (kcal/min)	Treadmill	1.25 m/s	53.29%
Price (2016)	One	Waist	Controlled	Energy Expenditure	Indirect calorimetry (TrueOne 2400 metabolic	EE (kcal/min)	Treadmill	1.80 m/s	46.23%
Price (2016)	One	Waist	Controlled	Energy Expenditure	Indirect calorimetry (TrueOne 2400 metabolic	EE (kcal/min)	Treadmill	2.22 m/s	48.71%
Price (2016)	One	Waist	Controlled	Energy Expenditure	Indirect calorimetry (TrueOne 2400 metabolic	EE (kcal/min)	Treadmill	2.78 m/s	29.98%
Price (2016)	One	Waist	Controlled	Energy Expenditure	Indirect calorimetry (TrueOne 2400 metabolic	EE (kcal/min)	Treadmill	3.33 m/s	14.41%
Reid (2016)	One	Bra	Freeliving	Steps	Accelerometry (Actigraph GT3X)	Steps / day	Normal Dality Activities	7 days (waking hours)	11.1%
Reid (2016)	One	Waist	Freeliving	Steps	Accelerometry (Actigraph GT3X)	Steps / day	Normal Dality Activities	7 days (waking hours)	11.1%
Reid (2016)	Flex	Wrist	Freeliving	Steps	Accelerometry (Actigraph GT3X)	Steps / day	Normal Dality Activities	7 days (waking hours)	6.4%
Reid (2016)	One	Waist	Freeliving	Time in Activity	Accelerometry (Actigraph GT3X)	Sedentary (Hours / day)	7 days (waking hours). FB Sleep	Sedentary 0-99 counts per minute	-15.3%

Reid (2016)	One	Bra	Freeliving	Time in Activity	Accelerometry (Actigraph GT3X)	Sedentary (Hours / day)	7 days (waking hours). FB Sleep	Sedentary 0-99 counts per minute	-13.3%
Reid (2016)	Flex	Wrist	Freeliving	Time in Activity	Accelerometry (Actigraph GT3X)	Sedentary (Hours / day)	7 days (waking hours). FB Sleep	Sedentary 0-99 counts per minute	-11.7%
Reid (2016)	One	Waist	Freeliving	Time in Activity	Accelerometry (Actigraph GT3X)	Light (Mins / Day)	7 days (waking hours)	Light 100-2019 counts per min	54.6%
Reid (2016)	One	Bra	Freeliving	Time in Activity	Accelerometry (Actigraph GT3X)	Light (Mins / Day)	7 days (waking hours)	Light 100-2019 counts per min	59.0%
Reid (2016)	Flex	Wrist	Freeliving	Time in Activity	Accelerometry (Actigraph GT3X)	Light (Mins / Day)	7 days (waking hours)	Light 100-2019 counts per min	4.6%
Reid (2016)	One	Waist	Freeliving	Time in Activity	Accelerometry (Actigraph GT3X)	Moderate (Min / Day),	7 days (waking hours)	Moderate 2020-5998 counts/min	42.1%
Reid (2016)	One	Bra	Freeliving	Time in Activity	Accelerometry (Actigraph GT3X)	Moderate (Min / Day),	7 days (waking hours)	Moderate 2020-5998 counts/min	31.6%
Reid (2016)	Flex	Wrist	Freeliving	Time in Activity	Accelerometry (Actigraph GT3X)	Moderate (Min / Day),	7 days (waking hours)	Moderate 2020-5998 counts/min	47.4%
Reid (2016)	One	Waist	Freeliving	Time in Activity	Accelerometry (Actigraph GT3X)	Vigorous (Min / Day),	7 days (waking hours)	Vigorous > 5999 counts/min	346.2%
Reid (2016)	One	Bra	Freeliving	Time in Activity	Accelerometry (Actigraph GT3X)	Vigorous (Min / Day),	7 days (waking hours)	Vigorous > 5999 counts/min	404.0%
Reid (2016)	Flex	Wrist	Freeliving	Time in Activity	Accelerometry (Actigraph GT3X)	Vigorous (Min / Day),	7 days (waking hours)	Vigorous > 5999 counts/min	409.4%
Sasaki (2015)	Classic	Hip	Controlled	Energy Expenditure	Indirect calorimetry (Oxycon Mobile)	Total EE (kcal / 6-min)	ADL	Raking	-28.95%
Sasaki (2015)	Classic	Hip	Controlled	Energy Expenditure	Indirect calorimetry (Oxycon Mobile)	Total EE (kcal / 6-min)	ADL	Vacumming	-11.73%
Sasaki (2015)	Classic	Hip	Controlled	Energy Expenditure	Indirect calorimetry (Oxycon Mobile)	Total EE (kcal / 6-min)	ADL	Laundry	-34.21%
Sasaki (2015)	Classic	Hip	Controlled	Energy Expenditure	Indirect calorimetry (Oxycon Mobile)	Total EE (kcal / 6-min)	ADL	Gardening	-11.73%
Sasaki (2015)	Classic	Hip	Controlled	Energy Expenditure	Indirect calorimetry (Oxycon Mobile)	Total EE (kcal / 6-min)	ADL	Office Work	-7.84%
Sasaki (2015)	Classic	Hip	Controlled	Energy Expenditure	Indirect calorimetry (Oxycon Mobile)	Total EE (kcal / 6-min)	ADL	Dusting	9.33%
Sasaki (2015)	Classic	Hip	Controlled	Energy Expenditure	Indirect calorimetry (Oxycon Mobile)	Total EE (kcal / 6-min)	Physical Activity: Stationary Bike	Cycling (300 kgm·min–1) * pacing?	-67.56%
Sasaki (2015)	Classic	Hip	Controlled	Energy Expenditure	Indirect calorimetry (Oxycon Mobile)	Total EE (kcal / 6-min)	Walk: Flat ground	Walking Carrying groceries - self selected speed	43.93%
Sasaki (2015)	Classic	Hip	Controlled	Energy Expenditure	Indirect calorimetry (Oxycon Mobile)	Total EE (kcal / 6-min)	Sport	Tennis	-19.59%
Sasaki (2015)	Classic	Hip	Controlled	Energy Expenditure	Indirect calorimetry (Oxycon Mobile)	Total EE (kcal / 6-min)	Sport	Golf	-3.76%
Sasaki (2015)	Classic	Hip	Controlled	Energy Expenditure	Indirect calorimetry (Oxycon Mobile)	Total EE (kcal / 6-min)	Sport	Basketball	15.91%
Sasaki (2015)	Classic	Hip	Controlled	Energy Expenditure	Indirect calorimetry (Oxycon Mobile)	Total EE (kcal / 6-min)	Stairs	Stairs - self selected speed	-20.81%
Sasaki (2015)	Classic	Hip	Controlled	Energy Expenditure	Indirect calorimetry (Oxycon Mobile)	Total EE (kcal / 6-min)	Walk Incline:Treadmill	4.0 mph, 5% incline [1.79 m/s]	-19.00%
Sasaki (2015)	Classic	Hip	Controlled	Energy Expenditure	Indirect calorimetry (Oxycon Mobile)	Total EE (kcal / 6-min)	Walk Inclinc:Treadmill	3.0 mph, 5% incline [1.34 m/s]	-26.39%
Sasaki (2015)	Classic	Hip	Controlled	Energy Expenditure	Indirect calorimetry (Oxycon Mobile)	Total EE (kcal / 6-min)	Jog:Treadmill	Jog 5.5 mph [2.46 m/s]	-2.74%
Schaffer (2017)	Zip	Waist	Controlled	Steps	Visual Observation / Count	Time: steps/6 min	Walking: Treadmill	6 MWT, self-paced, 70 or 100 feet (all) n=24	-11.02%
Simpson (2015)	One	Ankle	Controlled	Steps	Visual Observation / Count	Distance: Steps / 15 m	Walk: Hallway	15 m walk, 0.8m/s	0.00%
Simpson (2015)	One	Ankle	Controlled	Steps	Visual Observation / Count	Distance: Steps / 15 m	Walk: Hallway	15 m walk, 0.9m/s	3.57%
Simpson (2015)	One	Hip	Controlled	Steps	Visual Observation / Count	Distance: Steps / 15 m	Walk: Hallway	15 m walk, 0.8m/s	-6.67%
Simpson (2015)	One	Hip	Controlled	Steps	Visual Observation / Count	Distance: Steps / 15 m	Walk: Hallway	15 m walk, 0.9m/s	-3.57%
Simpson (2015)	One	Ankle	Controlled	Steps	Visual Observation / Count	Distance: Steps / 15 m	Walk: Hallway	15 m walk, 0.3m/s	-12.24%
Simpson (2015)	One	Ankle	Controlled	Steps	Visual Observation / Count	Distance: Steps / 15 m	Walk: Hallway	15 m walk, 0.6m/s	-3.03%
Simpson (2015)	One	Ankle	Controlled	Steps	Visual Observation / Count	Distance: Steps / 15 m	Walk: Hallway	15 m walk, 0.4m/s	0.00%
Simpson (2015)	One	Ankle	Controlled	Steps	Visual Observation / Count	Distance: Steps / 15 m	Walk: Hallway	15 m walk, 0.7m/s	0.00%
Simpson (2015)	One	Ankle	Controlled	Steps	Visual Observation / Count	Distance: Steps / 15 m	Walk: Hallway	15 m walk, 0.5m/s	2.86%
Simpson (2015)	One	Hip	Controlled	Steps	Visual Observation / Count	Distance: Steps / 15 m	Walk: Hallway	15 m walk, 0.3m/s	-97.96%
Simpson (2015)	One	Hip	Controlled	Steps	Visual Observation / Count	Distance: Steps / 15 m	Walk: Hallway	15 m walk, 0.4m/s	-82.93%
Simpson (2015)	One	Hip	Controlled	Steps	Visual Observation / Count	Distance: Steps / 15 m	Walk: Hallway	15 m walk, 0.5m/s	-40.00%
Simpson (2015)	One	Hip	Controlled	Steps	Visual Observation / Count	Distance: Steps / 15 m	Walk: Hallway	15 m walk, 0.6m/s	-21.21%
Simpson (2015)	One	Hip	Controlled	Steps	Visual Observation / Count	Distance: Steps / 15 m	Walk: Hallway	15 m walk, 0.7m/s	-13.33%
Stackpool (2014)	Ultra	Hip	Controlled	Steps	Visual Observation / Count	Time: Steps / 20 min	Physical Activity - Elliptical	Elliptical, self selected intensity (~12-13 of 20 Borg scale)	-0.04%
Stackpool (2014)	Ultra	Hip	Controlled	Steps	Visual Observation / Count	Time: Steps / 20 min	Run: Treadmill	Run self selected pace 20 minutes	-6.03%
Stackpool (2014)	Ultra	Hip	Controlled	Steps	Visual Observation / Count	Time: Steps / 20 min	Walk: Treadmil	Walk self selected pace 20 minutes	0.00%
Stackpool (2014)	Ultra	Hip	Controlled	Steps	Visual Observation / Count	Time: Steps / 20 min	Physical Activity - Agility	Sport Agility Activities (Ladder and Basketball) 20 minutes	-19.88%
Stackpool (2014)	Ultra	Hip	Controlled	Energy Expenditure	Indirect calorimetry (Oxycon Mobile)	Total EE (kcal / 10-min)	Physical Activity: Elliptical	Elliptical, self selected intensity (~12-13 of 20 Borg scale)	-4.35%
Stackpool (2014)	Ultra	Hip	Controlled	Energy Expenditure	Indirect calorimetry (Oxycon Mobile)	Total EE (kcal / 10-min)	Sport	Agility Activities (Ladder and Basketball)	-16.67%
Stackpool (2014)	Ultra	Hip	Controlled	Energy Expenditure	Indirect calorimetry (Oxycon Mobile)	Total EE (kcal / 10-min)	Walk: Treadmill	walking, self selected pace	1.83%
Stackpool (2014)	Ultra	Hip	Controlled	Energy Expenditure	Indirect calorimetry (Oxycon Mobile)	Total EE (kcal / 10-min)	Run: Treadmill	Run self selected pace	-4.17%
Sushames (2016)	Flex	Wrist	Controlled	Steps	Visual Observation / Count	Time: Steps/ 6 min	Incline Walking: Treadmill	5% incline, 1.39-1.8 m/s, self-paced (Borg's rate of perceived exhaustion	-8.21%

Sushames (2016)	Flex	Wrist	Controlled	Steps	Visual Observation / Count	Time: Steps/ 6 min	Jog: Treadmill	8-10 km/hr or 2.22-2.78 m/s, self-paced (Borg's rate of perceived [illegible]	2.52%
Sushames (2016)	Flex	Wrist	Controlled	Steps	Visual Observation / Count	Time: Steps/ 6 min	Walking: Treadmill	1.39-1.8 m/s, self-paced (Borg's rate of perceived exhaustion 4)	-14.68%
Sushames (2016)	Flex	Wrist	Controlled	Steps	Visual Observation / Count	Time: Steps/ 6 min	Stair stepping	15 cm box	1.07%
Takacs (2014)	One	Hip	Controlled	Steps	Visual Observation / Count	Time: Steps / 5 min	Walk: Treadmill	Walk 5 minutes 0.90 m/s	-0.25%
Takacs (2014)	One	Hip	Controlled	Steps	Visual Observation / Count	Time: Steps / 5 min	Walk: Treadmill	Walk 5 minutes1.54 m/s	-0.18%
Takacs (2014)	One	Hip	Controlled	Steps	Visual Observation / Count	Time: Steps / 5 min	Walk: Treadmill	Walk 5 minutes1.12 m/s	0.00%
Takacs (2014)	One	Hip	Controlled	Steps	Visual Observation / Count	Time: Steps / 5 min	Walk: Treadmill	Walk 5 minutes 1.78 m/s	0.00%
Takacs (2014)	One	Hip	Controlled	Steps	Visual Observation / Count	Time: Steps / 5 min	Walk: Treadmill	Walk 5 minutes 1.33 m/s	0.19%
Takacs (2014)	One	Hip	Controlled	Distance	Treadmill Distance	Distance: km / 5 min	Walk: Treadmill	Walk 5 minutes 0.90 m/s	40.0%
Takacs (2014)	One	Hip	Controlled	Distance	Treadmill Distance	Distance: km / 5 min	Walk: Treadmill	Walk 5 minutes1.12 m/s	26.9%
Takacs (2014)	One	Hip	Controlled	Distance	Treadmill Distance	Distance: km / 5 min	Walk: Treadmill	Walk 5 minutes 1.33 m/s	15.2%
Takacs (2014)	One	Hip	Controlled	Distance	Treadmill Distance	Distance: km / 5 min	Walk: Treadmill	Walk 5 minutes1.54 m/s	2.5%
Takacs (2014)	One	Hip	Controlled	Distance	Treadmill Distance	Distance: km / 5 min	Walk: Treadmill	Walk 5 minutes 1.78 m/s	-4.3%
Thorup (2017)	Zip	Waist	Freeliving	Steps	Accelerometer [Shimmer 3 (ankle)]	Steps / day	Normal Daility Activities	24 hours, waking hours (Hospital Ward)	-43%
Thorup (2017)	Zip	Waist	Freeliving	Steps	Accelerometer [Shimmer 3 (ankle)]	Steps / day	Normal Daility Activities	24 hours, waking hours	-25%
Treacy (2017)	One	Ankle	Controlled	Steps	Visual Observation / Count	Time: Steps / 6 min	Walking: Track (120 m)	6 MWT, self-paced, with or without walking aide	-16.00%
Treacy (2017)	One	Waist	Controlled	Steps	Visual Observation / Count	Time: Steps / 6 min	Walking: Track (120 m)	6 MWT, self-paced, with or without walking aide	-48.00%
Treacy (2017)	Charge	Wrist	Controlled	Steps	Visual Observation / Count	Time: Steps / 6 min	Walking: Track (120 m)	6 MWT, self-paced, with or without walking aide	-56.00%
van Blarigan (2017)	One	Waist	Freeliving	Steps	Accelerometry (Actigraph GT3X))	Steps / day	Normal Daility Activities	7 days, waking hours	9%
van Blarigan (2017)	One	Waist	Freeliving	Steps	Pedometry (Omron)	Steps / day	Normal Daility Activities	7 days, waking hours	36%
van Blarigan (2017)	One	Waist	Freeliving	Time in Activity	Accelerometry (Actigraph GT3X))	Light (Mins / Day)	8 days (waking hours)	AG:: 100-2019; FB < 3 METS (Light)	52%
van Blarigan (2017)	One	Waist	Freeliving	Time in Activity	Accelerometry (Actigraph GT3X))	Moderate (Min / Day),	9 days (waking hours)	AG: 2020-5998; 3-5.9 METs (Farily Active)	72%
van Blarigan (2017)	One	Waist	Freeliving	Time in Activity	Accelerometry (Actigraph GT3X))	Vigorous (Min / Day)	10 days (waking hours)	AG: > 5999; FB >= 6 METS (Very Active)	375%
van Blarigan (2017)	One	Waist	Freeliving	Time in Activity	Accelerometry (Actigraph GT3X))	MVPA (Mins / Day)	11 days (waking hours)	AG: > = 2020, FB > = 3 METS	96%
Wallen (2016)	Charge HR	Wrist	Controlled	Energy Expenditure	Indirect calorimetry (MetaMax 3B)	Total EE (Kcal / 58 min)	Physical Activity: Progressive [illegible]	Sedentary (lying, sitting, standing) and physical Activity (walking,cycling).	-20.83%
Wong (2017)	Ultra	Waist	Controlled	Steps	Visual Observation / Count	Time: steps/1 min	Walking: Treadmill	steps/4 min, 0.9 m/s	-29.95%
Wong (2017)	Ultra	Waist	Controlled	Steps	Visual Observation / Count	Time: steps/1 min	Walking: Treadmill	steps/4 min, 1.1 m/s	-5.14%
Wong (2017)	Ultra	Waist	Controlled	Steps	Visual Observation / Count	Time: steps/1 min	Walking: Treadmill	steps/4 min, 1.3 m/s	-5.88%
