# Supplementary material for: Accuracy of Fitbit Devices: Systematic Review and Narrative Syntheses of Quantitative Data
Source: JMIR Mhealth Uhealth. 2018 Aug 9;6(8):e10527. doi: 10.2196/10527 (PMC6107736; doi:10.2196/10527)
Supplement: Multimedia Appendix 4 [file mhealth_v6i8e10527_app4.pdf]

Modified COSMIN criteria used for Risk of Bias Assessment

**Design Requirements**	**Criterion**	**Excellent**	**Good**	**Fair**	**Poor**
Q1. Was the percentage of missing Fitbit/Criterion data given?		Percentage of missing data described- number of participants in both Fitbit and reference groups included in the analyses provided; relative to total number of participants in the study (i.e. It is clear that 28 of 30 participants in the study provided data for FB and reference measures)	Percentage of missing data NOT described- just total number of participants included in analysis (no individual group numbers), or no indication of how many including in the analyses at all (i.e. only the number of participants in the study is reported, no indication how many were included in the analyses and if any measures for either the FB or references were missing)		

Modified COSMIN criteria used for Risk of Bias Assessment

Q2. Was there a description of how missing data were handled?		Described how missing data were handled – describe explicitly why data is missing and how they dealt with the missing data statistically (i.e. only data with both FB and reference were included in the analyses, or all available data was included in the analyses)	Not described but it can be deduced how missing items were handled – do not state explicitly how missing data was dealt with, but can be deduced from table that less data was included in analysis for each group than total number of participants in study	Not clear how missing items were handled-no information, for example table show missing data but no explanation	
Q3. Was the sample size included in the analysis adequate?		Adequate sample size (≥ 100)	Good sample size (50-99)	Moderate sample size (30-49)	Small sample size (<30)
Q4. Can the criterion used or employed be considered as a reasonable 'gold standard'?	**Lab:**<br>Steps: VO<br>EE: IC or DC<br>Sleep: PSG<br>Distance: laser, tape measure, treadmill<br>Time: stopwatch(s)	Criterion used can be considered an adequate 'gold standard' (evidence provided)	No evidence provided, but assumable that the criterion used can be considered an adequate 'gold standard' -not calibrated treadmill if applicable	Unclear whether the criterion used can be considered an adequate 'gold standard'	Criterion used can NOT be considered an adequate 'gold standard' (i.e. self-reported time in activity is not a valid reference criterion)

Modified COSMIN criteria used for Risk of Bias Assessment

	**Free-Living** Accelerometer, except for EE, it should be doubly labelled water or steps could also be pedometer or sleep could be portable monitor				
Q5. Were there any important flaws in the design or methods of the study?		No other important methodological flaws in the design or execution of the study-Both wearables need to be on body at the same time, gold standard used, else adequate reference		Other minor methodological flaws in the design or execution of the study; meets criteria for E, but (e.g.) not talking about how they blocking/randomizing ADL's, no need to randomize activities in lab (should be the same), other differences in the way study was executed compared to similar study	Other important methodological flaws in the design or execution of the study – not meeting criteria under E
Q6. For continuous scores: Were correlations, AUC or BA plots?		Percent difference AND equivalency OR BA Plot OR MAPE%/SE of means, RMSE, CV, CCC	Percent difference only		No PD or way to calculate PD, but has other measures for accuracy (BA plot, MAPE/SE of means, RMSE, CV, CCC)
