# Supplementary material for: Accuracy of Fitbit Devices: Systematic Review and Narrative Syntheses of Quantitative Data
Source: JMIR Mhealth Uhealth. 2018 Aug 9;6(8):e10527. doi: 10.2196/10527 (PMC6107736; doi:10.2196/10527)
Supplement: Multimedia Appendix 5 [file mhealth_v6i8e10527_app5.pdf]

Data Coding – Steps and Energy Expenditure in Controlled Settings.

All comparisons in Steps and EE in controlled settings were coded for each of the following domains outlined below unless otherwise indicated as EE or Steps only				
**Type of Test**	**Speed**	**Type of 'Ambulation'**	**Limb (Body) Motion**	**Body Position**
Resting / Sitting [Si] – EE Only	Jog (≥ 2 m/s) [J]	Continuous No Incline [Cn]	Normal [Nr]	Torso [To]- Hip, chest, waist, or upper arm
Activity – EE Only	Normal (1-2 m/s) [Nm]	Intermittent [I] - No Incline	Constrained [C] (e.g. holding / pushing something, including walker, cane or constrained motion from disease such as stroke)	Wrist [Wr]
	Slow (0.8-0.9 m/s) [S]	Continuous Incline [Ci]	Exaggerated [Ex] (e.g. tennis, golf, carrying lap top bag)	Ankle/Foot [AF] - Steps Only
	Very Slow (<0.8 m/s) [Vs]		Mixed [Mi] (e.g. ADL. Gym, Sport or variable activities)	
	Self-Paced (Comfortable) [Sp]			
