# Supplementary material for: Accuracy of Fitbit Devices: Systematic Review and Narrative Syntheses of Quantitative Data
Source: JMIR Mhealth Uhealth. 2018 Aug 9;6(8):e10527. doi: 10.2196/10527 (PMC6107736; doi:10.2196/10527)
Supplement: Multimedia Appendix 6 [file mhealth_v6i8e10527_app6.pdf]

Included Sutdies - Study Characteristics												
First Author - Last Name	Pub Year (Mth)	Type	Country	Size	Age [Mean (SD)], Other	Gender	Health / Mobility Status	Fitbit Model(s)	Body Placement	Setting	Outcome	Criterion
Adam Noah	2014 (Oct)	A	USA	23	26.6 (7.55)	F=10, M=13	Healthy	Ultra, Classic	Hip	L	EE	ID
Alhrabi	2016 (Feb)	A	AUS	48	65.6 (6.9)	F=23, M=25	Cardiac, Bone-joint, Resp.	Flex	Wrist	F	Steps	Accel
											TiA	Accel
Alinia	2017 (Aug)	A	USA	15	Range 21-31	F=7, M=8,	Healthy	Zip, One, Flex	Chest, Waist, Wrist	L	Steps	VO
An	2017 (Mar)	A	USA	35	31.0 (11.8)	F=18, M=17	Healthy	Flex, Zip	Wrist, Waist	F	Steps	Ped
										L	Steps	VO
Bai	2016 (Jan)	A	USA	52	Range 18-60	F=24, M=28	Healthy	Flex	Wrist	L	EE	ID
Battenberg	2017 (Jun)	A	USA	30	25.6 (2.5)	F=12, M=18	Healthy	One, Force	Wrist, Waist	L	Steps	VO
Balto	2016 (Jan)	A	USA	45	46.7 (10.0)	nr	Multiple Sclerosis	Flex, One	Wrist, Waist	L	Steps	VO
Beevi	2015 (Oct)	A	DEN	14	29.9 (4.93)	F=5, M=9	Healthy	Zip	Hip	L	Steps	VO
Brewer	2017 (Sep)	A	USA	50	28.1 (9.12)	F=41, M=9	Healthy	Various	Arm	F	Steps	Accel
											TiA	Accel
Brooke	2017 (Apr)	A	USA	95	28.5 (9.9)	F=61, M=34	Healthy	Charge HR, Flex	Wrist	F	EE	Accel

											Sleep	Accel
Chen	2016 (May)	A	CHN	30	21.5 (2.0)	F=15, M=15	Healthy	Flex	Wrist	L	Steps	VO
Chowdhury	2017 (Feb)	A	UK	30	27 (6)	F=15, M=15	Healthy	Charge HR	Wrist	L	EE	ID
										F	EE	Accel
Chow	2017 (Sep)	A	AUS	31	24.3 (5.2)	F=12, M=19	Healthy	One, Flex, Charge HR	Waist, Wrist	L	Steps	VO
Chu	2017 (Feb)	A	USA	104	Med. 31.0	F=69, M=35	Healthy	Flex	Wrist	F	Steps	Accel
Cook	2017 (Aug)	A	USA	21	26.5 (4.6)	F=17, M=4	Depression	Flex	Wrist	L	Sleep	PSG
Dannecker	2013 (Nov)	A	USA	19	26.9 (6.6)	F=9, M=10	Healthy	Classic	Hip	L	EE	DD
Diaz	2016 (Apr)	R	USA	13	32.0 (9.2)	F=13, M=0	Healthy	One, Flex, Charge HR	Torso, Hip, Wrist	L	Steps	VO
											EE	ID
Dickinson	2017 (Nov)	A	USA	38	26.05 (8.0)	F=23, M=15	Healthy	Charge HR	Wrist	F	Sleep	Accel
Dominick	2016 (Sept)	A	USA	19	20.6 (nr)	F=15, M=4	Healthy	Flex	Wrist	F	Steps	Accel
Doolley	2017 (Mar)	A	USA	62	22.6 (4.3)	F=36, M=26	Healthy	Charge HR	Wrist	L	Energy Expendit ure	ID
Dondzila	2016 (Jun)	A	USA	19	24.6 (3.1)	F=5, M=14	Healthy	Charge HR	Wrist	L	EE	ID
Farina	2017 (Nov)	A	UK	25	72.5 (4.9)	F=13, M=12	Healthy	Charge HR	Wrist	F	Steps	Accel
Ferguson	2015 (Mar)	A	AUS	21	32.8 (10.2)	F=11, M=10	Healthy	One, Zip	Hip	F	Sleep	Accel

											EE	Accel
											Steps	Accel
											TiA	Accel
Floegel	2016 (June)	A	USA	99	78.9 (8.6)	F = 71, M= 29	Variable Mobility	One, Flex, Charge HR	Hip, Wrist	L	Steps	VO
Fokkema	2016 (Nov)	A	NED	31	32 (12)	F=15, M=16	Healthy	Charge HR	Wrist	L	Steps	VO
Fulk	2014 (Feb)	A	USA	50	52.9 (15.1)	F=16, M=34	Post-stroke	Ultra	Hip	L	Steps	VO
Gomersall	2016 (Sept)	A	AUS	29	39.6 (11.0)	F=26, M=3	Healthy	One	Waist	F	Steps	Accel
											TiA	Accel
Gusmer	2014 (Apr)	A	USA	32	21.2 (1.7)	F=25, M=7	Healthy	Ultra	Hip	L	EE	ID
Hargens	2017 (Jan)	A	USA	22	30.8 (11.4)	F=15, M=7	Healthy	Charge, One	Wrist, Waist	F	Steps	Accel
											EE	Accel
											TiA	Accel
Hui	2017 (Oct)	A	CAN	12	62.6 (9.3)	F=5, M=7	Post-stroke	One	Ankle (non-paretic)	F	Steps	Accel
											TiA	Accel
Husted	2017 (Jan)	A	USA	12	20.8 (0.94)	F=11, M=1	Healthy	Charge	Wrist	L	Steps	VO
Huang	2016 (Aprl)	A	CHN	40	23.9 (2.8)	F=10, M=30	Healthy	Zip, One, Flex	Wrist, Waist	L	Steps	VO

											Dist	Meas
Imboden	2017 (Apr)	A	USA	30	49.2 (19.2)	F=15, M=15	Healthy	One, Zip, Flex	Waist, Wrist	L	Steps	VO
											TiA	Accel
											EE	ID
Klassen	2015 (Oct)	A	CAN	43	65 (10.66)	F=13, M=30	Post-stroke	One	Hip, Ankle (non-paretic)	L	Steps	VO
Kooiman	2015 (Oct)	A	NED	33	35.3 (nr)	F=17, M=16	Healthy	Flex, Zip	Wrist, Hip	L	Steps	VO
										F	Steps	Accel
Lee	2014 (Sept)	A	USA	60	26.4 (nr)	F=30, M=30	Healthy	One, Zip	Hip	L	EE	IID
Lee	2017 (Oct)	A	KOR	16	22.8 (2.8)	F=10, M=6	Healthy	Charge HR	Wrist	F	Sleep	Accel
Madigan	2017 (Jan)	A	USA	30	80.6 (7.66)	F=18, M=12	Chronic Health Conditions	Flex	Wrist	L	Steps	VO
Mantua	2016 (May)	A	USA	40	22.37 (4.92)	F=19, M=21	Healthy	Flex	Wrist	L	Sleep	PSG
Middlewerd	2017 (Jun)	R	NED	34	23.9 (3.9)	F=23, M=11	Healthy	One	Hip	F	Steps	Accel
											TiA	Accel
Modave	2017 (Jun)	A	USA	60	49.5 (19.4)	F=36, M=24	Healthy	Surge	Wrist	L	Steps	VO
Montes	2017 (Oct)	R	USA	49	23.4 (6.6)	F=23, M=26	Healthy	Flex	Wrist	L	EE	ID
											Steps	VO

Montoye	2017 (Oct)	A	USA	32	23.5 (1.3)	F=14,M =18	Healthy	Charge HR	Wrist	L	EE	ID
Montgomery	2012 (Sep)	A	USA	24	26.1 (nr)	F=10, M=14	Healthy	Classic	Wrist	L	Sleep	PSG
Murakami	2016 (May)	L to Editor	JAP	19	Range 21-50	F=10, M=9	Healthy	Flex	Wrist	L	EE	DD
										F	EE	DLW
Nelson	2016 (Mar)	A	USA	30	48.9 (19.4)	F=15, M=15	Healthy	One, Zip, Flex	Hip, Wrist	L	Steps	VO
											EE	ID
O'Connell	2016 (May)	A	IRE	15	21.1 (1.1)	F=8, M=7	Healthy	One	Chest	L	Steps	VO
Park	2014 (May)	A	USA	40	38.6 (nr)	F=20, M=20	Healthy	Ultra	Chest, Hip, Wrist	L	Steps	VO
Paul	2015 (Jul)	A	AUS	32	67.7 (5.7)	F=20, M=12	Healthy	One, Zip	Hip	F	Steps	Accel
										L	Steps	VO
Phillips	2015 (Sep)	R	USA	50	84.2 (7.1)	F=45, M=5	Healthy	Classic	Hip	L	Steps	VO
Price	2016 (Dec)	A	AUS	14	23.0 (6.0)	F=3, M=11	Healthy	One	Waist or Bra	L	EE	VO
Reid	2016 (Nov)	A	CAD	22	21.23 (1.63)	F=22 M=0	Healthy	Flex, One	Wrist, Waist / Bra	F	Steps	Accel
											Time	Accel
Rosenberger	2016 (Mar)	A	USA	40	36, (nr)	F=21, M=19	Healthy	One	Hip, Wrist	F	Steps	Ped
											TiA	Accel
											Sleep	Monitor

Sasaki	2015 (Feb)	A	USA	20	24.1 (4.5)	F=10, M=10	Healthy	Classic	Hip	L	EE	ID
Schaffer	2017 (Oct)	A	USA	24	54 (13.4)	F=10, M=14	Post-stroke	Zip	Waist	L	Steps	VO
Scherbina	2017 (May)	A	USA	60	38.5 (nr)	F=31, M=29	Healthy	Surge	Wrist	L	EE	ID
Simpson	2015 (Oct)	A	CAN	42	73 (6.9)	F=31, M=11	Healthy	One	Hip, Ankle	L	Steps	VO
Stackpool	2014 (Dec)	A	USA	20	22 (nr)	F=10, M=10	Healthy	Ultra	NR	L	Steps	VO
											EE	ID
Sushames	2016 (Sept)	A	AUS	25	23.7 (5.8)	F=12, M=13	Healthy	Flex	Wrist	L	Steps	VO
Takacs	2014 (Sep)	A	CAN	30	29.6 (5.7)	F=15, M=15	Healthy	One	Hip	L	Steps	VO
											Dist.	Meas
Thorup	2017 (Mar)	A	DEN	44	53 (nr),	F=12, M=32	Healthy and Cardiac Disease	Zip	Hip, Chest	F	Steps	Ped
Treacy	2017 (May)	A	AUS	166	80 (11), 26-98	F=75, M=91	Inpatient Rehab Patients	One, Charge	Hip, Wrist, Ankle	L	Steps	VO
Tully	2014 (Dec)	R	UK	42	Med. 43	F=25, M=17	Healthy	Zip	Hip	F	EE	Accel
											Steps	Ped
Van Blarigan	2017 (Jan)	A	USA	22	Med. 66	F=0, M=22	Prostate Cancer Survivor	One	Waist	F	TiA	Accel
											Steps	Accel
Wahl	2017 (Sep)	A	GER	20	25.2 (nr)	F=10, M=10	Healthy		Wrist	L	Steps	VO

								Charge, Charge HR			Dist.	Meas
											EE	ID
Wallen	2016 (May)	A	AUS	22	24.9 (5.6)	F=11, M=11	Healthy	Charge HR	Wrist	L	EE	ID
											Steps	VO
Wong	2017 (Oct)	A	USA	25	25.96 (7.9)	F=12, M=13	Healthy	Ultra	Waist	L	Steps	VO
Type: (A)rticle, Short (R)eport, (L)etter to Editor												
Setting: (L)aboratory, (F)ree-Living												
Outcome: EE – Energy Expenditure, TiA – Time in Activity, Dist - Distance												
Criterion: Accel – Accelerometer, Ped – Pedometer, ID – Indirect Calorimetry, DD – Direct Calorimetry, VO – Visual Observation, Meas – Measured, PSG – Polysomnography, DLW – Doubly Labelled Water.												
