# Supplementary material for: Accuracy of Fitbit Devices: Systematic Review and Narrative Syntheses of Quantitative Data
Source: JMIR Mhealth Uhealth. 2018 Aug 9;6(8):e10527. doi: 10.2196/10527 (PMC6107736; doi:10.2196/10527)
Supplement: Multimedia Appendix 7 [file mhealth_v6i8e10527_app7.pdf]

Accuracy Evaluations Reported and Risk of Bias Assessment											
Study Information			Accuracy Evaluation(s) Reported			[b] *COSMIN* Risk of Bias Assessment					
First Author - Last Name	Setting	Outcome	[a] Mean / % Difference	Mean / Median Absolute Percent Error	LOA	Missing Data Report	Missing Data Handling	Sample Size	Acceptable Criterion	Design Flaws	Acceptable Accuracy
Adam Noah	C	EE	**Y**	N	N	G	F	P	E	E	G
Alhrabi	F	Steps	**Y**	N	Y	E	E	F	E	E	E
	F	TiA	**Y**	N	N	E	E	F	E	E	G
Bai	C	EE	**Y**	Y	Y	E	G	G	E	F	E
Balto	C	Steps	**Y**	N	N	G	F	F	E	E	E
Battenberg	C	Steps	**Y**	N	N	G	F	F	E	E	G
Beevi	C	Steps	**Y**	N	N	G	F	P	E	E	E
Brewer	F	Steps	**Y**	N	Y	E	E	G	E	E	E
	F	TiA	**Y**	N	Y	E	E	G	E	E	E
Brooke	F	EE	**Y**	Y	Y	E	E	P	E	E	E
	F	Sleep	**Y**	Y	Y	E	E	P	E	E	E
Chen	C	Steps	**Y**	Y	Y	G	F	F	E	E	E
Chow	C	Steps	**Y**	N	N	G	F	F	E	E	G
Chowdhury	C	EE	**Y**	Y	Y	G	F	F	E	E	E
Cook	C	Sleep	**Y**	N	Y	G	F	P	E	E	E
Dannecker	C	EE	**Y**	N	N	E	E	P	E	F	E
Diaz	C	Steps	**Y**	N	Y	G	F	P	E	E	E
	C	EE	**Y**	N	Y	G	F	P	E	E	E
Dominick	F	Steps	**Y**	N	N	E	E	P	E	E	G
Dondzila	C	EE	**Y**	Y	N	G	F	P	E	E	E
Doolley	C	EE	**Y**	Y	N	G	F	G	E	E	E
Farina	F	Steps	**Y**	N	Y	E	E	P	E	E	E
Ferguson	F	Sleep	**Y**	Y	Y	E	E	P	E	E	E
	F	EE	**Y**	Y	Y	E	E	P	G	E	E
	F	Steps	**Y**	Y	Y	E	E	P	E	E	E
	F	TiA	**Y**	Y	Y	E	E	P	E	E	E
Floegel	C	Steps	**Y**	Y	Y	E	E	G	E	E	E
Fokkema	C	Steps	**Y**	Y	Y	E	E	F	E	E	E
Fulk	C	Steps	**Y**	N	Y	E	E	G	E	E	E
Gomersall	F	Steps	**Y**	N	Y	E	E	P	E	E	E
	F	TiA	**Y**	**N**	Y	E	E	P	E	E	E
Gusmer	C	EE	**Y**	N	Y	G	F	F	E	E	E
Hargens	F	Steps	**Y**	Y	Y	G	G	P	E	E	E
Huang	C	Steps	**Y**	N	N	G	F	F	E	E	G
	C	Dist.	**Y**	N	N	G	F	F	E	E	G

Hui	F	Steps	**Y**	N	N	G	F	P	E	E	G
	F	TiA	**Y**	N	N	G	F	P	E	E	G
Imboden	C	Steps	**Y**	Y	Y	G	G	F	E	F	E
	C	TiA	**Y**	Y	Y	G	G	F	E	F	E
	C	EE	**Y**	Y	Y	G	G	F	E	F	E
Klassen	C	Steps	**Y**	N	N	E	E	F	E	E	E
Kooiman	C	Steps	**Y**	Y	Y	G	F	F	E	E	E
	F	Steps	**Y**	Y	Y	G	F	F	E	E	E
Lee (2014)	C	EE	**Y**	Y	Y	E	E	G	E	E	E
Lee (2017)	F	Sleep	**Y**	N	N	E	E	P	E	E	G
Mantua	C	Sleep	**Y**	Y	Y	E	E	F	E	E	E
Middlewerd	F	Steps	**Y**	N	Y	E	E	F	E	E	E
	F	TiA	**Y**	N	Y	E	E	F	E	E	E
Modave	C	Steps	**Y**	N	N	G	G	G	E	E	G
Montes	C	EE	**Y**	N	N	G	F	F	E	E	E
	C	Steps	**Y**	N	N	G	F	F	E	E	E
Montgomery	C	Sleep	**Y**	N	Y	G	F	P	E	E	E
Montoye	C	EE	**Y**	Y	Y	E	E	F	E	E	E
Murakami	C	EE	**Y**	N	N	G	F	P	E	E	G
	F	EE	**Y**	N	N	G	F	P	E	E	G
Nelson	C	Steps	**Y**	Y	N	E	E	F	E	F	E
	C	EE	**Y**	Y	N	E	E	F	E	F	E
O'Connell	C	Steps	**Y**	Y	Y	G	F	P	E	E	E
Park	C	Steps	**Y**	N	N	G	F	F	E	E	E
Paul	C	Steps	**Y**	N	Y	G	F	F	E	E	E
Phillips	C	Steps	**Y**	N	Y	E	E	G	E	E	E
Price	C	EE	**Y**	N	Y	G	F	P	E	E	E
Reid	F	Steps	**Y**	N	Y	E	E	P	E	E	G
	F	TiA	**Y**	N	Y	E	E	P	E	E	G
Sasaki	C	EE	**Y**	N	Y	G	F	P	E	F	E
Schaffer	C	Steps	**Y**	Y	Y	E	E	P	E	E	E
Simpson	C	Steps	**Y**	N	Y	G	F	F	E	E	E
Stackpool	C	Steps	**Y**	N	N	G	F	P	E	E	G
	C	EE	**Y**	N	N	G	F	P	E	E	G
Sushames	C	Steps	**Y**	N	Y	E	E	P	E	E	E
Takacs	C	Steps	**Y**	N	Y	G	F	F	E	E	E
	C	Dist.	**Y**	N	Y	G	F	F	E	F	E
Thorup	F	Steps	**Y**	N	Y	G	F	P	E	E	E
Treacy	C	Steps	**Y**	N	N	E	E	E	E	E	G
Van Blarigan	F	TiA	**Y**	N	N	E	E	P	E	E	G
	F	Steps	**Y**	N	N	E	E	P	E	E	G
Wallen	C	EE	**Y**	N	Y	E	E	P	E	E	E
Wong	C	Steps	**Y**	N	N	E	E	P	E	E	G

Supplementary Accuracy Studies (Or Outcomes) Excluded from Quantitative Analyses											
Alinia	C	Steps	N	Y	N	G	F	P	E	E	P
An	F	Steps	N	Y	Y	G	F	F	E	E	P
	C	Steps	N	Y	Y	G	F	F	E	E	P
Chowdhury	F	EE	N	Y	Y	G	F	F	E	E	P
Chu	F	Steps	N	Y	Y	E	E	E	E	E	P
Dickinson	F	Sleep	N	N	Y	E	E	F	E	E	P
Hargens	F	EE	N	Y	Y	G	G	P	G	E	P
	F	TiA	N	Y	Y	G	G	P	E	E	P
Husted	C	Steps	N	N	Y	G	F	P	E	E	P
Madigan	C	Steps	N	N	Y	E	E	F	E	E	P
Paul	F	Steps	N	N	Y	G	F	F	E	E	P
Rosenberger	F	Steps	N	Y	Y	G	F	F	E	E	P
	F	TiA	N	Y	Y	G	F	F	E	E	P
	F	Sleep	N	Y	Y	G	F	F	E	E	P
Scherbina	C	EE	N	Y	N	G	G	G	E	E	P
Tully	F	EE	N	N	Y	E	E	F	G	E	P
	F	Steps	N	N	Y	E	E	F	E	E	P
Wahl	C	Steps	N	Y	Y	G	F	P	E	E	P
	C	Dist.	N	Y	Y	G	F	P	E	E	P
	C	EE	N	Y	Y	G	F	P	E	E	P
Wallen	C	Steps	N	N	Y	E	E	P	E	E	P
Risk of Bias Summary					Excellent (n)	44	43	2	96	90	57
					Good (n)	55	9	10	3	0	21
					Excellent or Good (%)	100%	53%	12%	100%	91%	79%
					Fair (n)	0	43	39	0	9	0
					Poor (n)	0	0	46	0	0	21
					Fair or Poor (%)	0%	43%	86%	0%	9%	21%
[a] Group Mean or Percent Difference Reported = **Y**es (Included in Quantitative Analyses) N (Not included in Quantitative Analyses)											
[b] *COSMIN*: *CO*nsensus-based *S*tandards for the selection of health status *M*easurement *In*struments [16,17]											
C=Controlled, F=Free-Living, Dist. = Distance. TiA = Time in Activity. Y=Yes, N=No, E= Excellent, G=Good, F=Fair, P=Poor, LOA = Level of Agreement											
