# Supplementary material for: Accuracy of Fitbit Devices: Systematic Review and Narrative Syntheses of Quantitative Data
Source: JMIR Mhealth Uhealth. 2018 Aug 9;6(8):e10527. doi: 10.2196/10527 (PMC6107736; doi:10.2196/10527)
Supplement: Multimedia Appendix 8 [file mhealth_v6i8e10527_app8.pdf]

Controlled Settings: Accuracy – Measurement Error (%).									
Outcome	Sub-Categories	#	Measurement Error (%) - Within / Outside ± 3%			Measurement Error (%) - Point Estimates		Measurement Error (%) – Dispersion	
			Within ±3% [# (%)]	Less than -3% [# (%)]	Greater than 3% [# (%)]	Mean	Median	Minimum	Maximum
Steps - All Comparisons		191	88 (46%)	97 (51%)	6 (3%)	-9.3%	-3.3%	-98.0%	11%
Steps - Speed of Ambulation	Jog (> 2.0 m/s)	24	14 (58%)	10 (42%)	0	-4.7%	-1.8%	-42.2%	2.7%
	Normal (1.0-2.0 m/s)	48	25 (52%)	22 (46%)	1 (2%)	-3.5%	-2.4%	-14.7%	6.5%
	Self-paced (Comfortable)	70	32 (46%)	35 (50%)	3 (4%)	-10.9%	-3.3%	-63.5%	11.1%
	Slow (0.8-0.9 m/s)	23	10 (44%)	12 (52%)	1 (4%)	-5.1%	-3.4%	-30.0%	3.6%
	Very Slow (<0.8 m/s)	26	7 (27%)	19 (73%)	0	-24.1%	-12.2%	-98.0%	2.9%
Steps - Body Placement	Torso	114	65 (57%)	48 (42%)	1 (1%)	-10.6%	-2.0%	-98.0%	3.7%
	Wrist	61	15 (25%)	43 (70%)	3 (5%)	-8.6%	-6.3%	-57.4%	11.1%
	Ankle	16	8 (50%)	7 (44%)	1 (6%)	-2.9%	-1.1%	-16.0%	3.6%
Steps - Body Motion	Normal Motion	154	82 (53%)	70 (45%)	2 (2%)	-6.2%	-2.4%	-98.0%	6.5%
	Constrained Motion	24	5 (21%)	19 (79%)	0	-20.9%	-11.8%	-81.5%	0.0%
	Variable Motion	10	0	10 (100%)	0	-35.0%	-26.2%	-63.5%	-8.1%
	Exaggerated Motion	3	1 (33%)	0	2 (67%)	6.2%	7.5%	0.0%	11.1%
Energy Expenditure - Resting		10	3 (30%)	6 (60%)	1 (10%)	-3.1%	-5.6%	-15.1%	19.0%
Energy Expenditure - All Activities		88	4 (4%)	41 (47%)	43 (49%)	4.4%	2.3%	-67.6%	85.7%
Energy Expenditure - Body Placement	Torso	52	2 (4%)	32 (63%)	18 (35%)	-4.7%	-7.6%	-67.6%	53.3%
	Wrist	36	3 (8%)	9 (25%)	24 (67%)	17.6%	9.3%	-52.6%	85.7%
Energy Expenditure -	Jog (> 2.0 m/s)	15	2 (13%)	5 (33%)	8 (53%)	7.1%	4.6%	-22.5%	48.7%

Speed of Ambulation	Normal (1.0-2.0 m/s)	24	0	7 (29%)	17 (71%)	18.0%	11.8%	-41.4%	82.3%
	Self-paced (Comfortable)	39	2 (5%)	25 (64%)	12 (31%)	-6.4%	-8.9%	-67.6%	44.0%
	Slow (0.8-0.9 m/s)	6	0	2 (33%)	4 (68%)	15.3%	7.0%	-10.7%	85.7%
	Very Slow (<0.8 m/s)	4	1 (25%)	2 (50%)	1 (25%)	3.1%	-3.3%	-44.3%	63.3%
Energy Expenditure - Type of Ambulation	Continuous - No Incline	53	3 (6%)	15 (28%)	35 (66%)	16.7%	12.8%	-67.6%	85.7%
	Continuous -Incline	11	1 (9%)	7 (64%)	3 (27%)	-18.7%	-20.8%	-44.3%	10.0%
	Intermittent - No Incline	24	1 (4%)	18 (75%)	5 (21%)	-12.0%	-12.5%	-34.2%	29.0%
Energy Expenditure - Body Motion	Normal Motion	58	5 (9%)	19 (33%)	34 (59%)	12.2%	8.6%	-44.3%	30.0%
	Constrained Motion	10	0	6 (60%)	4 (40%)	-7.9%	-10.3%	-67.6%	44.0%
	Exaggerated Motion	4	0	3 (75%)	1 (25%)	-4.6%	-4.1%	-19.6%	9.3%
	Variable Motion	16	0	13 (81%)	3 (19%)	-13.7%	-14.6%	-34.2%	15.9%
Sleep - Sensitive Mode	Total Sleep	1	0	1 (100%)	0	-18.9%	n/a	n/a	n/a
	Sleep Efficiency	1	0	1 (100%)	0	-19.0%	n/a	n/a	n/a
	Sleep Onset Latency	1	0	0	1 (100%)	59.9%	n/a	n/a	n/a
	Wake After Sleep Onset	1	0	0	1 (100%)	109.5%	n/a	n/a	n/a
Sleep - Normal Mode	Total Sleep	3	1 (33%)	0	2 (67%)	10.1%	9.1%	3.0%	18.0%
	Sleep Efficiency	3	0	0	3 (100%)	12.9%	11.6%	8.8%	18.0%
	Sleep Onset Latency	1	0	1 (100%)	0	-12.0%	n/a	n/a	n/a
	Wake After Sleep Onset	1	0	1 (100%)	0	-181.0%	n/a	n/a	n/a
Distance - Torso	Slow (0.9 m/s)	3	0	0	3 (100%)	33.7%	31.2%	29.9%	40.0%
	Normal (1-1.5 m/s)	4	1 (25%)	0	3 (75%)	11.0%	9.4%	-1.8%	26.9%
	Brisk (1.5-2 m/s)	4	1 (25%)	3 (75%)	0.0%	-5.4%	-6.0%	-12.1%	2.5%
	Self-Paced	2	0	0	2 (100%)	4.8%	n/a	4.1%	5.4%

Distance - Wrist	Slow (0.9 m/s)	1	0	0	1 (100%)	26.8%	n/a	n/a	n/a
	Normal (1-1.5 m/s)	1	1 (100%)	0	0	-2.6%	n/a	n/a	n/a
	Brisk (1.5-2 m/s)	1	0	1 (100%)	0	-19.6%	n/a	n/a	n/a
	Self -Paced	1	0	0	1 (100%)	12.8%	n/a	n/a	n/a
