# Supplementary material for: Accuracy of Fitbit Devices: Systematic Review and Narrative Syntheses of Quantitative Data
Source: JMIR Mhealth Uhealth. 2018 Aug 9;6(8):e10527. doi: 10.2196/10527 (PMC6107736; doi:10.2196/10527)
Supplement: Multimedia Appendix 9 [file mhealth_v6i8e10527_app9.pdf]

Free-Living Settings: Relative Accuracy – Measurement Error (%).										
Outcome	Reference Criterion	Sub-Categories	#	Measurement Error (%) - Within / Outside +/- 10%			Measurement Error (%) – Point Estimates		Measurement Error (%) - Dispersion	
				Within ± 10% [# (%)]	Less than -10% [# (%)]	Greater than 10% [# (%)]	Mean	Median	Min	Max
Sleep (Min / Night)	Sensewear or Actiwatch	Wrist - Time in Bed	4	4 (100%)	0	0	4.0%	n/a	-0.4%	9.0%
	Actiwatch	Wrist - Total Sleep	1	1 (100%)	0	0	5.8%	n/a	n/a	n/a
Time in Activity (Min / Day)	Actical (ankle) or ActiGraph	Sedentary	4	1 (25%)	3 (75%)	0	-11.1%	-12.5%	-15.3%	-4.0%
		Light	5	1 (20%)	0	4 (80%)	43.5%	53.2%	4.6%	59.0%
		Moderate	6	0	1 (17%)	5 (83%)	40.6%	44.8%	-72.6%	122.6%
		Vigorous	6	0	0	6 (100%)	632%	389.5%	346%	1900%
	ActiGraph	MVPA	7	1 (29%)	0	6 (71%)	84.9%	96.1%	2.6%	150.0%
Steps (Steps / Day)	All Accuracy Comparisons		20	11 (55%)	3 (15%)	6 (30%)	7.5%	8.3%	-43.1%	30.2%
	ActiGraph	Torso (Old)	1	1 (100%)	0	0	9%	n/a	n/a	n/a
		Torso (Young)	7	5 (71%)	0	2 (29%)	8.4%	8.86%	4.4%	11.1%
		Wrist (Old)	2	0	1 (50%)	1 (50%)	-11%	n/a	-36%	13.0%
		Wrist (Young)	4	2 (50%)	0	2 (50%)	15.3%	12.3%	6.4%	30.2%
	ActivPAL	Wrist / Torso (Young)	2	2 (100%)	0	0	4.3%	n/a	1.2%	7.4%
	Omron	Torso (Old)	1	0	0	1 (100%)	36.3%	n/a	n/a	n/a
	Shimmer or Actical (ankle)	Torso (Old - Limited Mobility)	3	1 (33%)	2 (66%)	0	-24.3%	-25.3%	-43.1%	-4.7%

Energy Expenditure [Kcal / Day(s)]	Sensewear	Wrist / Torso	4	0	4 (100%)	0	-15.3%	-15.4%	-16.5%	-13.7%
	Doubly Labelled Water	Wrist	1	1 (100%)	0	0	-7.4%	n/a	n/a	n/a
MVPA = Moderate to Vigorous Physical Activity<br>Accelerometers = ActiGraph, Actical ActivPAL, Actiwatch or Sensewear<br>Pedometers = Omron or Shimmer										
